# New fluid biomarkers tracking non-amyloid-β and non-tau pathology in Alzheimer’s disease

**DOI:** 10.1038/s12276-020-0418-9

**Published:** 2020-04-13

**Authors:** Sun Ah Park, Song Mi Han, Chae Eun Kim

**Affiliations:** 1 Lab for Neurodegenerative Dementia, Department of Anatomy, Ajou University School of Medicine, Suwon, 16499 Republic of Korea; 2 Department of Neurology, Ajou University School of Medicine, Suwon, 16499 Republic of Korea; 3 Neuroscience Graduate Program, Department of Biomedical Sciences, Ajou University Graduate School of Medicine, Suwon, 16499 Republic of Korea

**Keywords:** Alzheimer's disease, Predictive markers, Alzheimer's disease, Predictive markers

## Abstract

Cerebrospinal fluid (CSF) biomarkers based on the core pathological proteins associated with Alzheimer’s disease (AD), i.e., amyloid-β (Aβ) and tau protein, are widely regarded as useful diagnostic biomarkers. However, a lack of biomarkers for monitoring the treatment response and indexing clinical severity has proven to be problematic in drug trials targeting Aβ. Therefore, new biomarkers are needed to track non-Aβ and non-tau pathology. Many proteins involved in the pathophysiological progression of AD have shown promise as new biomarkers. Neurodegeneration- and synapse-related biomarkers in CSF (e.g., neurofilament light polypeptide [NFL], neurogranin, and visinin-like protein 1) and blood (e.g., NFL) aid prediction of AD progress, as well as early diagnosis. Neuroinflammation, lipid dysmetabolism, and impaired protein clearance are considered important components of AD pathophysiology. Inflammation-related proteins in the CSF, such as progranulin, intercellular adhesion molecule 1, and chitinase-3-like protein 1 (YKL-40), are useful for the early detection of AD and can represent clinical severity. Several lipid metabolism-associated biomarkers and protein clearance-linked markers have also been suggested as candidate AD biomarkers. Combinations of subsets of new biomarkers enhance their utility in terms of broadly characterizing AD-associated pathological changes, thereby facilitating precise selection of susceptible patients and comprehensive monitoring of the treatment response. This approach could facilitate the development of effective treatments for AD.

## Alzheimer’s disease: New ways to track disease progression

Finding new biomarkers for Alzheimer’s disease (AD) may help in tracking disease progression and identifying optimal patient-specific treatments. Although useful markers are available for diagnosis of AD, they are unreliable for tracking disease progression. Looking for better ways to track disease progression, Sun Ah Park at the Ajou University School of Medicine, Suwon, South Korea, and coworkers have reviewed alternative AD markers. They report that several markers for axonal degeneration, synaptic loss, brain inflammation and lipid metabolism show promise for tracking AD. Some of these markers can be obtained from blood samples, which are minimally invasive to collect. Use of combinations of markers is especially promising for estimating a patient’s disease stage. These results will contribute to developing tailored treatments for this common cause of dementia.

## Introduction

Alzheimer’s disease (AD) is the most common neurodegenerative disorder that eventually results in dementia. The initial pathologic definition of AD constitutes accumulations of amyloid-β (Aβ) and pathologically modified tau proteins to form senile plaque and neurofibrillary tangles, respectively, which are regarded as core pathologic features in AD^1^. The measurements of Aβ_1-42_ (Aβ_42_), total tau (tTau), and phosphorylated tau at Thr181 (pTau_181_) in cerebrospinal fluid (CSF), as well as the visualization of fibrillar Aβ protein loads in the brain using a radioactive ligand, have proven useful in the early diagnosis of AD, which leads to their inclusion in diagnostic guidelines^2,3^ and biological definitions of AD^4^. Current clinical trials use measurements of Aβ protein and tau proteins in CSF and/or blood to guide participant recruitment and outcome measures^5^. This practice permits the enrollment of patients with AD pathology, even at the preclinical stage, and allows monitoring of treatment effects on Aβ- and tau-pathology. However, early saturation of Aβ accumulation in the brain, indicated by plateaus in CSF Aβ_42_ levels and amyloid PET uptake after clinical symptom onset, limits the usefulness of Aβ biomarkers for monitoring disease progression and drug response^6–8^. Tau protein levels are more likely than Aβ to reflect the clinical status; however, their clinical correlations are also lost with the advancement of neurodegeneration, revealing stabilization or a reduction in protein levels^8,9^. The shortage of Aβ and tau biomarkers is a serious problem during successive failures of Aβ-targeting drug trials. Treatment-responsive improvements in Aβ biomarkers (e.g., reduced Aβ uptake on amyloid PET and increased levels of CSF Aβ)^10–13^ and tau biomarkers^12^ were not accompanied by clinical benefits^13–16^. This finding clearly showed that changes in Aβ and tau biomarkers are not reliable in terms of predicting disease progression and monitoring clinical status. Therefore, new biomarkers are needed to resolve this shortage. Ideally, new biomarkers should represent Aβ- and tau-independent AD pathology, thereby enabling monitoring of clinical and biological benefits during Aβ- and tau-targeting therapies, in which changes in Aβ- and tau-biomarkers are inevitable.

The heterogeneity of AD is a considerable obstacle for the development of efficient disease-modifying treatments and the establishment of ideal disease-tracking biomarkers. Large neuropathological studies have demonstrated that pure AD pathology is infrequent in elderly patients with cognitive decline^17^. The precise pathophysiology, which determines the probability of developing clinical symptoms of dementia, is diverse in terms of the presence of Aβ and tau pathology. The actual contribution of AD pathology to cognitive loss has been estimated to vary from 22 to 100%^17^. This suggests that Aβ and tau pathologies alone cannot sufficiently represent the clinical severity of AD. Considerations of other biomarkers that directly signify pathologic substrates indicative of cognitive dysfunction are necessary.

## Overview of biological targets for non­-Aβ and ­-tau biomarkers

**Fig. 1:**
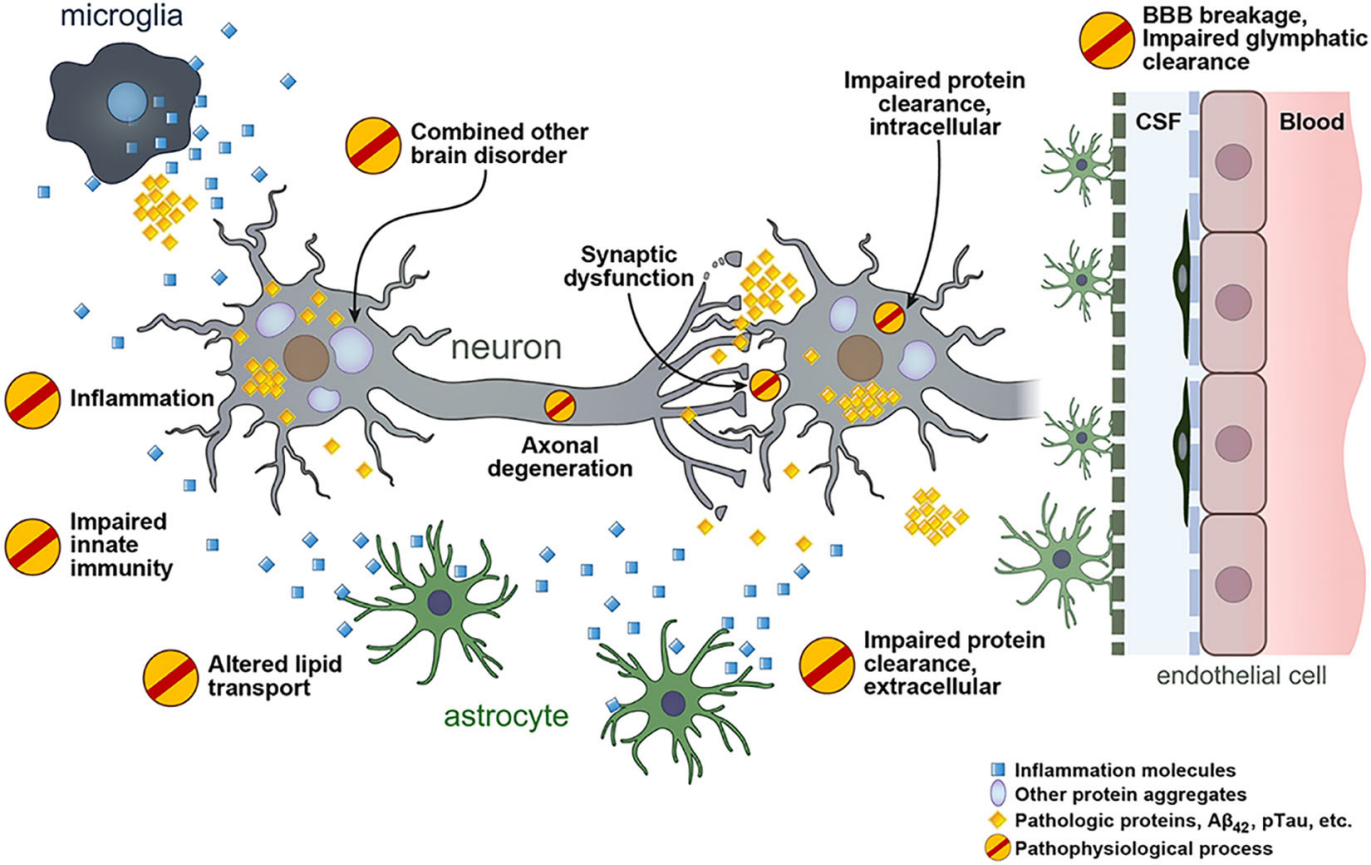
Overview of the pathophysiological process in Alzheimer’s disease. BBB, blood–brain barrier; CSF, cerebrospinal fluid.

Proteins related to various aspects of AD pathophysiological progression have been suggested as new fluid biomarkers in AD (Fig. 1). In addition to senile plaque and neurofibrillary tangles, dystrophic axons and dendrites surrounded by activated glial cells are abundant in the AD brain, which directly represent neurodegeneration and synapse loss^18^. Synapse loss is closely correlated with cognitive dysfunction^18^. Thus, neurodegeneration-related biomarkers may most closely indicate cognitive status. However, these biomarkers have received minimal attention in the field of AD biomarker development because they represent nonspecific and event-ending pathologies that are commonly observed in many neurodegenerative disorders. Nonetheless, accumulating evidence supports their clinical usefulness in diagnosis and clinical staging^19–23^.

Although astrogliosis and neuroinflammation are prevalent features in the AD brain^18^, the roles of microglia and astrocytes in AD pathophysiology have received less attention than neurons. However, many AD-related risk genes identified in genome-wide association studies (e.g., *ABCA7, CD33, CR1, EPHA1, MS4A*, and *TREM2*) are reportedly expressed in microglia and involved in neuroinflammation^24^. Accumulating experimental data support the active role of neuroinflammation in AD pathogenesis; moreover, it is currently regarded as a target for the development of AD treatments^25^. Various markers signifying the activation of inflammatory brain cells and the release of neuroinflammation-modulating factors have been suggested as potential biomarkers.

The *APOE* ε4 allele is the strongest and most prevalent risk gene for AD^24^. The primary biological role of the apolipoprotein E (ApoE) protein is to transport lipids and regulate cholesterol metabolism^26^. Lipid homeostasis is important in the physiological functions of the brain, including cellular membrane function, synaptic integrity, neuronal regeneration, and neuronal plasticity^27^. The disturbed lipid metabolism in AD is evidenced by many lipid droplets within glial cells^28^, as well as altered lipid content and distribution^29^, which are expected to contribute to AD pathogenesis. Proteins involved in lipid metabolism have been suggested as biomarkers for the diagnosis and monitoring of disease progression in AD.

Late and sporadic onset occurs in more than 90% of patients with AD. While synthesis of Aβ is the primary problem in AD with genetic mutation, impaired abnormal protein clearance is the main pathogenesis in sporadic AD^30^. Inside neurons and other brain cells, abnormal protein burdens are diminished by the autophagy–lysosomal system, ubiquitin–proteasome system and chaperone-mediated autophagy to maintain intracellular homeostasis^31^. In the extracellular space, protein clearance is mediated via protease, phagocytosis by astrocytes and microglia and exportation through the glymphatic system and blood–brain barrier (BBB) into CSF and systemic circulation^31^. Therefore, checking protein degradation machinery-related proteins might provide information regarding disease status caused by abnormal protein accumulation.

The frequent coexistence of vascular pathology and other neurodegenerative disorders, such as TDP-43 (e.g., frontotemporal dementia) and α-synuclein proteinopathy (e.g., Parkinson’s disease, Lewy body dementia, and multiple system atrophy), makes the pathophysiology and clinical manifestations of AD more variable^17^. The identification of combined brain pathology is necessary to correctly estimate AD status and predict disease progression because enhanced neurodegeneration can result in augmented cognitive dysfunction when other brain disorders are added. Several fluid biomarkers specific to core pathologic proteins of other neurodegenerative disorders, such as TDP-43 and α-synuclein protein levels, have emerged as potential biomarkers in frontotemporal dementia and Lewy body dementia/Parkinson’s disease dementia, respectively^32,33^. This review does not extend to non-AD-specific biomarkers because these have not yet yielded consistent results^32,34^. Instead, the differential diagnostic values of new biomarkers are discussed in the context of AD vs. non-AD pathologies.

**Fig. 2:**
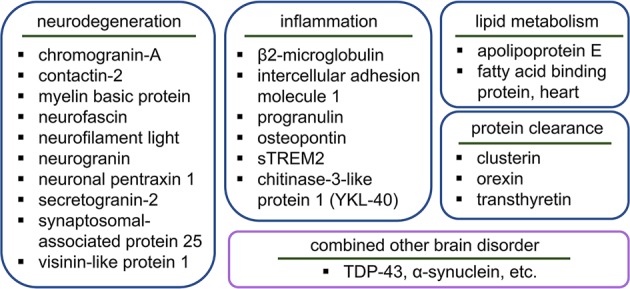
Overview of candidate non-Aβ and non-tau fluid biomarkers.

To develop novel biomarkers, two types of approaches have been conducted in large studies: targeted and nontargeted. The targeted approach uses hypothesis-driven methodology to verify candidate proteins that are preselected following basic experiments and bioinformatics analyses. In contrast, the nontargeted approach is purely data-driven. If candidate biomarkers are suggested by an explorative study, a subsequent validation study is needed using targeted measurement to confirm the validities of the biomarkers. This review is focused on new biomarkers (e.g., non-Aβ and non-tau biomarkers) for which the significance is supported by two or more independent studies involving different cohorts. Based on the most relevant biological pathways, candidate biomarkers are grouped and described for easy understanding (Fig. 2).

## Neurodegeneration-related biomarkers: synaptic loss and axonal degeneration

Proteins that exhibit changing expression in CSF and blood during the development and progression of AD are valuable as disease-tracking biomarkers. Because neurodegeneration is inevitable in AD and increases with AD progression, many synapse- and axon-related protein levels in CSF or blood have been closely investigated in many studies. Several proteins have been identified as promising AD biomarkers (Table 1 and Supplementary Table 1).

**Table 1:** Utility of new fluid biomarkers in Alzheimer’s disease.

	Early diagnosis	Specific diagnosis	Prediction	Correlation
*Neurodegeneration-related*				
Chromogranin-A (CSF)	↑MCI vs. CON ↓/→AD vs. CON	NA	NA	? Brain atrophy
Contactin-2 (CSF)	↑MCI vs. CON ↑/↓AD vs. CON	NA	NA	? Cognition
Myelin basic protein (CSF)	↑AD vs. CON	NA	NA	NA
Neurofascin (CSF)	↑MCI vs. CON ↓AD vs CON	NA	NA	NA
Neurofilament light (CSF)	↑Preclinical AD vs. CON ↑MCI vs. CON ↑AD vs. CON	Not specific	Cognitive decline Brain atrophy	Cognition Brain atrophy & hypometabolism
Neurofilament light (blood)	↑preclinical AD vs. CON ↑preclinical MC vs. NC ↑MCI vs. CON ↑AD vs. CON	Not specific	Cognitive decline Brain atrophy	Cognition Brain atrophy
Neurogranin (CSF)	↑preclinical AD vs. CON ↑MCI vs. CON ↑AD vs. CON	Specific to AD	Cognitive decline Brain atrophy	Cognition Brain atrophy
Neurogranin (NDE in blood)	↓/→AD vs. CON	? Not specific	? Cognitive decline in MCI	NA
Neuronal pentraxin 1 (CSF)	↑MCI vs. CON ↓AD vs. CON	NA	NA	NA
Secretogranin-2 (CSF)	↑MCI vs. CON ↓AD vs. CON	NA	? Cognitive decline in MCI	NA
SNAP-25 (CSF)	↑MCI-Aβ (+) vs. CON ↑AD vs. CON	NA	NA	NA
VILIP-1 (CSF)	↑MCI vs. CON in most ↑AD vs. CON in most	Possible	Cognitive decline	Brain atrophy
*Inflammation-related*				
β2-microglobulin (CSF)	↑MCI vs. CON	NA	? Cognitive decline in MCI	NA
ICAM1 (CSF)	↑ preclinical AD vs. CON ↑MCI vs. CON ↑AD vs. CON	NA	? Cognitive decline & its rapidity	Cognition
Progranulin (CSF)	↑MC vs. NC ↑AD vs. preclinical AD	Not specific	NA	Cognition Brain atrophy Brain hypometabolism
Osteopontin (CSF)	↑MCI vs. CON ↑AD vs. CON	Controversial	Cognitive decline	? Acuteness of cognitive dysfunction
sTREM2 (CSF)	↑/→ preclinical AD vs. CON ↑MCI vs. CON ↑peak at MCI > AD > CON ↑AD vs. CON	Not specific	NA	Age No association with cognitive function
YKL-40 (CSF)	↑preclinical AD vs. CON ↑MCI vs. CON ↑AD vs. CON ↑AD vs. MCI	Not specific	Maybe cognitive decline	Maybe cognition Gray matter atrophy Advancement of disease stage
YKL-40 (blood)	↑AD vs. CON	Not specific	NA	? Cognition Age
*Lipid metabolism-related*				
Apolipoprotein E (CSF)	↓/↑AD vs. CON ↓AD vs. MCI	Controversial	Cognitive decline in *APOE*ε4 noncarriers	Brain atrophy in *APOE*ε4 noncarriers
FABP3 (CSF)	↑MCI vs. CON ↑AD vs. CON	Controversial	Cognitive decline in MCI	Cognition Brain atrophy
*Protein clearance-related*				
Clusterin (CSF)	↑AD vs. CON	? Not specific	NA	? Cognition
Clusterin (blood)	→ AD vs. CON	NA	NA	? Cognition & brain atrophy
Orexin (CSF)	↑MCI vs. CON ↑/→AD vs. CON	? Possible	NA	NA
Transthyretin (CSF)	↑/→AD vs. CON	Controversial	NA	NA
Transthyretin (blood)	↓AD vs. CON	NA	NA	? Rapidity & severity of cognitive decline

*AD* Alzheimer’s disease, *CON* control, *CSF* cerebrospinal fluid, *FABP-3* fatty acid binding protein, heart, *ICAM1* intercellular adhesion molecule 1, *MC* mutation carrier, *MCI* mild cognitive impairment, *NA* not applicable due to lack of evidence, *NC* noncarrier, *NDE* neuron-derived exosome, *SNAP-25* synaptosomal-associated protein 25, *sTREM2* soluble triggering receptor expressed on myeloid cells 2, *VILIP-1* visinin-like protein 1, *YKL-40* chitinase-3-like protein 1.

↑, increased protein level; ↓, decreased protein level; →, no change in protein level; ?, not sure due to insufficient number of studies.

### Neurofilament light polypeptide

Neurofilament light polypeptide (NFL) is the most abundant component of large myelinated axons, which is released into CSF and systemic circulation when neurodegeneration occurs^35^. NFL has been extensively examined in terms of its clinical utility, and many studies have demonstrated its high degree of usefulness in clinical applications (Supplementary Table 1). It has also been documented that CSF NFL levels are well correlated with plasma NFL levels, although the levels in plasma are 50-fold lower than those in CSF^19,36,37^. Both CSF and plasma NFL levels are increased in relation to AD progression, revealing a high degree of correlation with cognitive functions and a good predictive value for future cognitive decline^20–22,36^. However, in terms of the differentiation of AD from other neurodegenerative disorders, NFL levels are less likely to be beneficial. High plasma NFL levels were frequently observed in progressive supranuclear palsy^38^, frontotemporal dementia^39^, multiple system atrophy, and corticobasal degeneration^40^. The capability of plasma NFL levels to predict cognitive decline and cortical regional atrophy was noticeable in both progressive supranuclear palsy and in AD^38^. Therefore, NFL levels are currently regarded as representative of neurodegeneration itself, independent of Aβ and tau pathology, which can be useful for proper disease tracking in both AD and non-AD dementia. Increases in NFL levels are evident well before the clinical onset of cognitive impairments: a distinct increase in serum NFL in AD-causative mutation carriers was observed 16.2 years earlier than clinical symptoms began, according to the findings of the Dominant Inherited Alzheimer’s Disease Network study^19^. In a study of normal elderly individuals without cognitive impairment, increased CSF NFL levels were observed in those who developed cognitive decline during follow-up^9,22^. The measurement of NFL in body fluids, especially easily accessible blood, is therefore expected to be applicable for preventative screening of preclinical stages of AD.

### Neurogranin

Neurogranin is a postsynaptic protein that is abundant in dendritic spines and plays a role in synaptic activity and plasticity. In contrast to NFL, which represents axonal degeneration, neurogranin signifies synaptic degeneration. In cross-sectional comparisons, many reports have demonstrated that neurogranin increases in CSF from patients with AD and patients with mild cognitive impairment (MCI) relative to that from healthy controls^41–44^ (Supplementary Table 1). Furthermore, increased CSF neurogranin is specific to AD among the various neurodegenerative disorders^23,43,44^. The value of CSF neurogranin for the prediction of future cognitive decline was also identified, although the direction differed among studies. Many studies have suggested that increased baseline neurogranin levels are indicative of future cognitive deterioration in patients with MCI^20,41,42^, while a few studies have suggested that low baseline neurogranin levels are indicative of future cognitive deterioration^22^. This discrepancy may be caused by dynamic changes in CSF neurogranin levels, depending on disease stage^9^, which has also been demonstrated for other synapse-related proteins (i.e., an early increase above and subsequent gradual reduction below the levels of controls, corresponding to disease progression)^45^. The early transient increase in CSF level is presumably due to the active degradation of synapses and compensatory enlargement of the remaining synapses^18^. Measurements of neurogranin levels in peripheral blood were performed, including neuron-derived exosomes; reduced levels of neurogranin in neuron-derived exosomes were reported in patients with AD compared with controls, but further validation is needed^46^.

### Visinin-like protein 1

Visinin-like protein 1 (VILIP-1) is a neuronal calcium sensor protein that is exclusively expressed at high levels in neurons^47^. Its release into CSF and systemic circulation is regarded as a marker of neuronal injury. Increased levels of VILIP-1 in CSF have often been found in patients with AD compared with healthy controls and patients with other neurodegenerative disorders, such as Lewy body dementia, frontotemporal dementia, and progressive supranuclear palsy; this finding suggests that increased VILIP-1 levels may constitute a specific marker for AD (Supplementary Table 1). However, no differences in VILIP-1 levels between patients with AD and controls^48^ or between patients with AD and those with vascular dementia or frontotemporal dementia have been reported^49^. Considering that longitudinal reduction in VILIP-1 levels occurs with disease progression after the initial increase in AD^9^, the increased levels of VILIP-1 in CSF could be unclear at certain stages of advanced clinical disease, which could weaken the validity of VILIP-1 as a biomarker. Therefore, stage-dependent interpretation of VILIP-1 levels is needed. At early stages of AD, such as preclinical and MCI stages, high CSF VILIP-1 levels predict future cognitive decline^48,50^ and brain atrophy^51^.

### Other candidate neurodegeneration-related biomarkers

Chromogranin-A and secretogranin-1 (also known as chromogranin-B) are well-known soluble components of large dense-core vesicles that play critical roles in the formation of secretory vesicles. Granin proteins are involved in various biological functions, including vasodilation, antiapoptosis, mast cell migration, microglial activation, neurotransmitter release, and synaptic function^52^. Altered CSF levels of granins are reportedly correlated with brain regional atrophy in patients with AD^45,53,54^. Chromogranin-A and secretogranin-1 exhibit characteristically dynamic changes in CSF expression according to disease stage in a manner similar to that of neurogranin: increased levels during early stages (i.e., MCI) and reduced levels during advanced dementia. Contactin-2 organizes the Ranvier nodes of axons and cell adhesion and was identified as a potential CSF biomarker in AD^55^. Myelin basic protein has a role in the formation and maintenance of the myelin sheath and was identified as a candidate CSF biomarker in AD and subcortical vascular disease^56^. Proteins involved in neurite outgrowth and synaptic stabilization, such as neurofascin and neuronal pentraxin 1, were suggested as CSF AD biomarkers^45,54^. Synaptosomal-associated protein 25, which has a role in neurotransmitter release, was also suggested as a candidate CSF biomarker^9^. These synapse-related proteins have the potential to be valuable disease-tracking biomarkers, as well as diagnostic biomarkers, considering that synapse loss is the factor most representative of clinical severity^18^.

## Neuroinflammation-related biomarkers

Modulators of inflammation and markers of activated inflammatory cells have been suggested as new biomarkers in AD (Table 1 and Supplementary Table 2).

### β2-Microglobulin and intercellular adhesion molecule 1

β2-Microglobulin is involved in the innate immune system through antigen presentation to the immune system^57^. Intercellular adhesion molecule 1 (ICAM1) is a cell-surface glycoprotein in endothelial cells and immune cells, which provides ligands that facilitate adhesion of leukocytes to endothelial cells; this allows leukocyte trafficking into the brain^58^. Both β2-microglobulin and ICAM1 in CSF are reportedly increased in patients with AD at the early, preclinical, and MCI stages^45,59^. Moreover, ICAM1 levels in CSF have been correlated with the severity of cognitive decline^60^.

### Progranulin

Progranulin (encoded by the *GRN* gene) is a growth factor that is expressed in neurons and microglia and is released from these cells. Progranulin is involved in neuroinflammatory modulation, specifically toward reducing microgliosis and astrogliosis^61^; moreover, it enhances neuronal outgrowth and neuronal survival^62^. Its expression is highly increased during microglial activation and neuronal maturation. Because *GRN* gene mutations are pathogenic with respect to the development of frontotemporal dementia spectrum disorders, the relation of progranulin with AD has received less attention. However, clinical manifestations of *GRN* mutations can also extend to AD^63^. The possibility of using progranulin as an AD biomarker was recently investigated in a large population of patients with familial and late-onset sporadic AD (Dominant Inherited Alzheimer’s Disease Network and Alzheimer’s Disease Neuroimaging Initiative cohorts)^64^. The CSF levels of progranulin were reportedly increased 10 years before the clinical onset of disease in AD mutation carriers. In patients with sporadic AD, increased CSF levels of progranulin were evident when neurodegeneration developed^64^. Increased levels of progranulin in CSF were also detected in suspected non-AD pathophysiology (SNAP) cases (i.e., normal Aβ biomarkers despite abnormalities in tau or neurodegeneration biomarkers; A−/TN+^4^)^64^. However, correlations of CSF progranulin levels with cognitive functions and CSF tau protein levels were present only in patients with AD, not in patients with SNAP^64^.

### Soluble triggering receptor expressed on myeloid cells 2 (sTREM2)

sTREM2 is an ectodomain of triggering receptor expressed on myeloid cells 2 (TREM2) that is released following proteolytic cleavage by α-secretases, disintegrin and metalloproteinase domain-containing protein 10 (ADAM10) and ADAM17^65^. TREM2 is expressed on the surface of microglia and is involved in innate immunity through modulation of microglial activity^66^. Several loss-of-function genetic variants of TREM2 have been shown to increase the risk of AD, including a variant at the His157 site, which affects the rate of release of sTREM2 into the extracellular space^65,67^. The role of sTREM2 in the progression of AD pathogenesis is under active investigation; it is potentially involved in modulating the survival and activity of microglia^68,69^. The released sTREM2 can be measured in both CSF and blood. Most studies have shown that increased levels of CSF sTREM are indicative of AD; however, a few studies showed no change in these levels in patients with AD (Supplementary Table 2). Longitudinal measurement in AD mutation carriers revealed altered CSF levels 5 years before expected clinical onset, when signs of brain amyloidosis and neurodegeneration were already obvious^70^. CSF sTREM2 levels were highest at the clinical stage of MCI, compared with other stages of AD^71,72^; increases in these levels were most pronounced immediately before the onset of dementia symptoms, when widespread neurodegeneration and synaptic loss were ongoing. Alterations in CSF sTREM2 levels were also identified in patients with other brain disorders, as well as in patients with SNAP^71^. Thus, there may not be a specific relationship between sTREM2 and AD. Extensive blood measurements of sTREM2 have rarely been performed; in the few studies involving these measurements, differences based on AD diagnosis were not evident, and whether there was a correlation with CSF sTREM2 levels was unclear^73^. A recent longitudinal follow-up study in Japan demonstrated that high serum sTREM2 levels were associated with future overall development of dementia, rather than AD or vascular dementia^74^. Additional studies with more refined measurement tools are needed to elucidate the precise value of blood sTREM2 levels as a biomarker for AD.

### Chitinase-3-like protein 1 (YKL-40)

YKL-40 is a carbohydrate-binding protein that is secreted by activated macrophages and microglia. It is thought to be involved in the modulation of inflammation, migration of astrocytes, and remodeling of tissue. Increased expression of YKL-40 has mainly been identified in reactive astrocytes in various neurological disorders, including AD, which suggests that YKL-40 is important in the astrocyte response to disease-related environmental conditions^75^. Elevated YKL-40 levels in CSF have often been identified and suggested to represent AD-related increased inflammation and astrocytosis in the earlier stages of AD, such as MCI or subclinical disease (Supplementary Table 2). However, there may not be a specific relationship between YKL-40 levels and Aβ pathology because no differences in YKL-40 levels have been detected between patients with AD and patients with other neurodegenerative dementias, such as Lewy body dementia, vascular dementia, and frontotemporal dementia. The progression of clinical symptoms and brain cortical atrophy are more closely associated with increases in YKL-40 levels^22,76,77^. Therefore, increases in YKL-40 are presumably linked to a common pathway that results in neurodegeneration itself, rather than a specific disease process.

### Other candidate inflammation-related biomarkers

In addition to the above candidates, other inflammation-related proteins have been suggested as possible biomarkers. Osteopontin is a glycophosphoprotein with roles in cell-matrix interactions and innate immunity. It is involved in inflammatory processes as a proinflammatory cytokine and modulates the activity of immune cells such as macrophages and microglia^78^. Increased CSF osteopontin levels have been reported in patients with AD and MCI compared with healthy controls (Supplementary Table 2). Larger increases in CSF osteopontin may represent disease progression and acute-phase disease^79^. However, reports have been contradictory in terms of specificity for AD. Levels of various complement proteins^59^, including fms-related tyrosine kinase^60^, fractalkine^72^, interleukin-10^80^, interleukin-15^60^, lysozyme C^45^, macrophage migration inhibitory factor^53^ and monocyte chemoattractant protein 1^72^, are significantly altered in patients with AD. These findings should be further explored using refined measurement tools and large sample sizes of patients with AD at various stages to confirm their usefulness as AD biomarkers.

## Lipid metabolism-related biomarkers

Lipid transport is essential for neuronal survival, synaptic activity and immune responses of glial cells in the brain^81^. Proteins linked to lipid metabolism have been suggested as candidate AD biomarkers (Table 1 and Supplementary Table 3). ApoE is involved in lipid homeostasis through regulation of the production, conversion, and clearance of lipoprotein, as well as in lipid transport via lipidation and subsequent binding to cell-surface receptors (e.g., LDL receptor family members)^82^. The presence of ApoE4 isoforms is known to increase AD risk due to the altered physiological function of the ApoE protein in the brain^82^. Measurements of ApoE protein levels in CSF have been performed in relation to the diagnosis of AD; notably, contradictory results have been demonstrated (Supplementary Table 3). ApoE protein levels have been suggested for use in the differential diagnosis of AD from Lewy body dementia and other disorders; however, further validation is needed due to the lack of supporting evidence.

Heart fatty acid-binding protein (FABP3) is another lipid-binding protein that plays a role in lipid transport. FABP3 is released from myocytes during the early stages of myocardial infarction; thus, blood levels of FABP3 constitute a useful biomarker for early diagnosis of heart attack^83^. In patients with AD, FABP3 levels in CSF have been reported to increase as early as the MCI stage (Supplementary Table 3). Higher baseline levels of FABP3 in patients with MCI could predict conversion to AD during follow-up^84^. FABP3 levels exhibit a weak ability to discriminate AD from other brain disorders involving dementia. However, consideration of pTau_181_ CSF levels in combination with FABP3 levels has been shown to increase the accuracy of differentiating AD from Lewy body dementia^85^.

Based on the results of a nonbiased proteomic study and subsequent quantitative selective reaction monitoring that included small numbers of CSF samples from patients with AD, Parkinson’s disease, Lewy body dementia, and nonneurodegenerative conditions, levels of other lipid metabolism-linked proteins (e.g., beta-2-glycoprotein 1 [also known as apolipoprotein H], ectonucleotide pyrophosphatase/phosphodiesterase family member 2 [also known as autotaxin], prosaposin, and vitamin D-binding protein) were reportedly increased in the CSF of patients with AD and have been suggested as possible biomarkers in AD^86^.

## Biomarkers related to the clearance of neurotoxic proteins

Proteins related to the degradation and removal of abnormal proteins, Aβ and pathologic tau proteins, have been suggested as candidate biomarkers (Table 1 and Supplementary Table 4). Clusterin is a secretory glycoprotein, is mainly produced by astrocytes in the brain^87^, and serves as a molecular chaperone; it binds to partially unfolded proteins, thereby preventing their aggregation^88^. Clusterin also has a neuronal differentiation-promoting effect and neuroprotective properties^87^. Missense and small deletion polymorphisms in the clusterin gene increase the risk of AD^24^; these findings suggest that it plays a role in AD pathology. In CSF, levels of clusterin were reported to increase in patients with AD and Lewy body dementia and were correlated with cognitive decline.

Orexin is a neuropeptide that regulates circadian rhythm and related physiological homeostasis^89^. Its reduction in CSF is a well-established biomarker for narcolepsy. In relation to AD, altered levels of orexin in CSF have generally been reported to increase, with a few exceptions (Supplementary Table 4). Furthermore, infusion of orexin from patients with AD led to increased Aβ production and amyloid deposition in human *AβPP* transgenic mice carrying the Swedish mutation, which suggested that it has a pathophysiological role in AD^90^.

Transthyretin is an Aβ-binding molecule that has been reported to inhibit Aβ aggregation, thereby reducing Aβ-induced cellular toxicity^91^. It has been reported to increase or remain stable in CSF, whereas it has been reported to decrease in blood and in patients with AD who exhibited rapid and severe cognitive decline (Supplementary Table 4). In addition, cystatin C, GM2 ganglioside activator, LAMP-1, ubiquitin, ubiquitin carboxyl-terminal esterase L1, carboxypeptidase E, carnosine dipeptidase 1, and ectonucleotide pyrophosphatase/phosphodiesterase were found to be higher in the CSF of patients with AD than in that of controls^86^. Matrix metalloproteinase proteins are potentially altered in the CSF of patients with AD^56^.

## The combinations of new biomarkers

The simultaneous consideration of new biomarkers is beneficial in concurrent assessment of various aspects of AD pathophysiology, thereby correctly estimating disease status^92^. There have been multiple recent investigations to establish reliable and useful combinations of CSF biomarkers (Table 2). The co-consideration of neurodegeneration and inflammation markers, neurogranin and YKL-40, improved the accuracy of differential diagnosis of AD from non-AD dementia, achieving an area under the receiver operating characteristic curve of 85%^93^. The co-consideration of neurogranin and NFL levels (neurogranin represents synaptic damage while NFL reflects axonal damage) demonstrated improved diagnostic accuracy of AD relative to that of each marker alone^20^; both proteins showed significant predictive associations with cognitive decline and brain atrophy. However, NFL levels were significantly associated with cognitive decline and brain atrophy in all patients, regardless of amyloid pathology, while neurogranin levels were significantly associated with cognitive decline and brain atrophy only in patients with amyloid pathology^20^. When both biomarkers were compared directly, NFL levels were superior to neurogranin as a prognostic biomarker in MCI patients with positive Aβ biomarker results^94^ and in normal elderly individuals (mean age, 59.3 ± 6.3 years)^95^.

**Table 2:** Sets of new biomarker combinations.

Combination	Findings
CSF neurogranin, YKL-40^93^	▪ Both increased in AD, but not correlated with each other: represent different pathology in AD ▪ Higher differential diagnostic value of neurogranin than YKL-40, AD vs. non-AD (85% of AUC)
CSF chromogranin-A, FABP-3, matrix metalloproteinase-2, pancreatic polypeptide levels + regional brain volume on MRI + CSF Aβ_42_, pTau_181_, tTau levels^53^	▪ Improved accuracy in the prediction of MCI conversion to AD on 12 m FU (95% accuracy) when combined
CSF neurogranin, NFL + CSF tTau levels^20^	▪ Improved diagnostic accuracy in AD vs. CON when combined (neurogranin, NFL, tTau), showing highest AUC (85.5%) ▪ tTau and neurogranin: strongly associated with cognitive decline and brain atrophy in case of Aβ (+) on 2 yr FU ▪ NFL: associated with cognitive decline and brain atrophy independent of Aβ pathology, in Aβ (+)/(−) on 2 yr FU
CSF FABP-3, IL-10, NFL^80^	▪ Different pattern over longitudinal change: (1) increased FABP-3: more sensitive to milder AD stages, (2) increased IL-10: associated with rate of longitudinal cognitive decline at MCI stage, (3) increased NFL: most strongly associated with the dementia stage of AD ▪ These are complementary to each other in AD clinical staging
CSF neurogranin, NFL^94^	▪ NFL: highest accuracy in prediction of MCI conversion to AD compared to neurogranin, Aβ_42_, pTau_181_, and tTau levels, on >1 yr FU
CSF neurogranin, SNAP-25, VILIP-1, YKL-40^9,98^	▪ Different pattern in longitudinal change, on 1-7 yr FU-LP ▪ Complementary to each other in AD clinical staging ▪ Combination of baseline Aβ_42,_ neurogranin, SNAP-25, VILIP-1 and YKL-40; combination of baseline pTau, neurogranin and SNAP-25: good correlation with baseline cognition in MC ▪ Combination of baseline Aβ_42_, tTau, neurogranin, SNAP-25 and VILIP-1: prediction of EYO
CSF neurogranin, NFL + CSF tTau levels^95^	▪ NFL: stronger correlation with cognitive decline at FU than neurogranin and tTau levels
CSF clusterin, fractalkine, MCP-1, sTREM2, YKL-40^71^	▪ All increased in subjects with neurodegeneration ▪ Different starting time of level change: (1) sTREM2 from subclinical stage, (2) MCP-1 from MCI stage, (3) YKL-40 and clusterin from dementia stage
CSF neurogranin, NFL, YKL-40 + CSF tTau^22^	▪ Different prediction accuracy of cognitive decline depending on clinical stage (2.3 yr FU at mean): (1) in CON-Aβ (+) group: high baseline NFL levels predicts cognitive decline. (2) in MCI-Aβ (+) group: high baseline NFL and tTau and decreased neurogranin levels can predict cognitive decline. (3) in AD-Aβ (+) group: increased baseline NFL and neurogranin levels can predict cognitive decline. (4) in MCI-Aβ (−) group: increased baseline NFL and tTau levels can predict cognitive decline.

*AD* Alzheimer’s disease, *AUC* area under the curve, *CON* control, *CSF* cerebrospinal fluid, *EYO* expected year of onset of AD in mutation carrier, *FABP-3* fatty acid binding protein, heart, *FU* follow-up, *IL-10* interleukin-10, *LP* lumbar puncture, *m* month, *mc* mutation carrier, *MCI* mild cognitive impairment, *MCP-1* monocyte chemoattractant protein 1, *MRI* magnetic resonance imaging, *NFL* neurofilament light polypeptide, *SNAP-25* synaptosomal-associated protein 25, *sTREM2* soluble triggering receptor expressed on myeloid cells 2, *tTau* total tau protein, *pTau* phosphorylated tau protein, *VILIP-1* visinin-like protein 1, *YKL-40* chitinase-3-like protein 1, *yr* year.

Aβ (+)/(−), positive (+) or negative (−) Aβ biomarker, either on CSF or amyloid positron emission tomography (PET).

Combinations of synaptic degeneration markers (e.g., neurogranin, synaptosomal-associated protein 25, and VILIP-1) with the inflammation marker YKL-40 revealed a remarkable and differential longitudinal change across the clinical spectrum of AD patients^9^. This finding suggests that specific biomarkers become more useful at particular disease stages^9,72,80^. Therefore, synaptic, axonal, and inflammation-related biomarkers are complementary to each other in terms of staging AD and predicting clinical progression, in addition to representing different aspects of AD-related brain pathology.

## Perspective on the utility of new biomarkers

### Proper disease-tracking and clinical trial design

The staging of AD has mainly relied on clinical data, cognitive impairment, and activities of daily living, which can divide AD into the following stages: preclinical AD, MCI due to AD (or prodromal AD), and AD dementia^2,3^. Recently, the A/T/N system has provided more precise AD staging based on AD-related pathological processes, i.e., the deposition of Aβ (A) and abnormal tau (T) proteins and neurodegeneration (N)^4^. For clinical application of the A/T/N system, sufficient evidence supporting the utility of CSF and neuroimaging biomarkers is needed, including CSF Aβ42, the CSF Aβ42/Aβ40 ratio and amyloid PET for A; CSF pTau and Tau PET for T; and structural MRI, fluorodeoxyglucose PET and CSF tTau levels for N^4^. Since its introduction, new biomarkers to support the A/T/N system have been required^4^. For N, which relies on CSF tTau levels (which can be affected by tau pathology), the identification of new biomarkers of neurodegeneration, such as CSF NFL and neurogranin levels, may now be imminent. In many studies, CSF NFL better reflected clinical severity and predicted future cognitive decline more accurately than Aβ and tau proteins^22,94,95^. Therefore, CSF NFL could improve our ability to track disease progression.

**Fig. 3:**
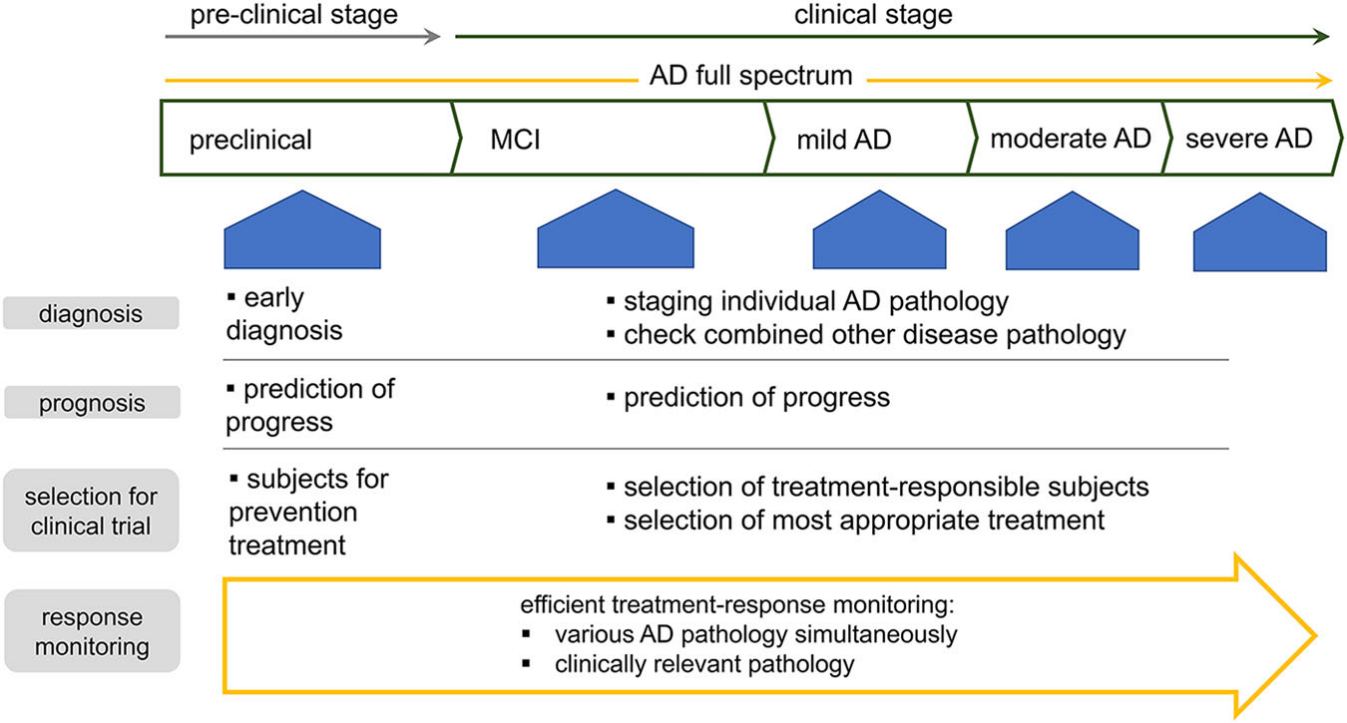
Perspectives on future clinical utility of new biomarkers. AD, Alzheimer’s disease; MCI, mild cognitive impairment.

Characterizing AD-related pathophysiology through biomarkers linked to diverse aspects of AD pathophysiology (e.g., neurodegeneration, synaptic dysfunction, neuroinflammation, lipid dysmetabolism and disturbed protein clearance) would be helpful for predicting the progression of individual facets of the pathology and for understanding their relative contributions to clinical deterioration^96^. A comprehensive understanding of disease status will aid in the selection of patients who are most likely to have a favorable response to specific disease-modifying therapies. Since other brain disorders commonly cooccur with AD^7^, considering both disease-specific and more general biomarkers of pathology may increase the likelihood of realizing efficient disease-modifying therapeutics. This approach could lead to more efficient treatment regimens and allow monitoring of the response to treatment on an individual patient basis, which is important given the diversity of AD pathology. Such precision medicine can maximize the therapeutic effect (Fig. 3).

### Readily accessible blood biomarkers

CSF is continuous with the interstitial fluid of the brain and directly reflects chemical changes in the brain. Most fluid biomarkers that have proven valuable for the diagnosis or monitoring of AD have been measured in CSF. Lumbar puncture, which is necessary to obtain CSF, is generally safe and well tolerated; however, it is time consuming and sometimes results in postpuncture headache and other side effects^97^. There are increasing efforts to develop easily assessable fluid biomarkers (e.g., from peripheral sources). Specifically, blood levels of NFL have shown strong evidence of usefulness, as described above, which are well correlated with CSF levels of NFL. With advances in ultrasensitive measurement tools, additional useful biomarkers from peripheral sources are likely to be identified. These promising peripheral fluid biomarkers will aid in increasing the efficiency of clinical trials with fewer costs and difficulties and provide mass screening of early AD for prevention.

## Concluding remarks

The value of new biomarkers demonstrates that the consideration of diverse AD pathophysiology (e.g., other than Aβ- and tau-centered aspects, such as neurodegeneration, synaptic dysfunction, neuroinflammation, lipid dysmetabolism, and disturbed protein clearance) would help to develop useful disease-tracking AD biomarkers. Neurodegeneration-related biomarkers represent axonal injury, synaptic dysfunction, or synaptic loss. Early diagnostic and prognostic values of these markers are well established. However, in terms of the differentiation of AD from other brain disorders, synaptic proteins (e.g., neurogranin and VILIP-1) are useful, whereas axonal injury markers (e.g., NFL) are not. However, NFL is excellent for predicting disease progression and tracking disease severity. Therefore, co-consideration of NFL with AD-specific biomarkers may be complementary and increase clinical utility. Neuroinflammation is regarded as a main component of AD pathophysiology. CSF levels of ICAM1, progranulin, sTREM2, and YKL-40 are useful in early AD diagnosis. However, their specificities for AD are controversial, and disease-tracking characteristics are unclear for some biomarkers. In particular, dynamic changes in the levels of sTREM2 based on clinical stage make its value unclear. Stage-dependent changes in biomarkers are also prevalent among synaptic markers during longitudinal assessment, which complicates the clinical applications of these biomarkers; however, these changes provide clues that can be used for disease staging and tracking following appropriate interpretation. Lipid metabolism-related biomarkers (e.g., ApoE and FABP3) and several protein clearance-related markers may have advantages, but more convincing data are needed for some of them. Considering that these mechanisms are intimately related to AD pathogenesis and are candidate targets of disease-modifying AD therapeutics, some of these biomarkers are expected to be useful in the future after more evidence is obtained.

The ideal combination of new biomarkers for enhanced clinical application has not yet been determined; however, the simultaneous incorporation of neurodegeneration- and neuroinflammation-related biomarkers is likely to be optimal. For NFL, neurogranin, VILIP-1, YKL-40, and FABP3 levels in CSF and for NFL levels in blood, there is substantial evidence supporting their value as diagnostic and prognostic biomarkers in patients with AD. In the near future, some of these new biomarkers may be incorporated into diagnostic criteria and research frameworks for AD, in combination with the current Aβ and tau biomarkers, to improve the staging and prediction of disease progression in patients with AD. Consideration of new biomarkers that represent different pathological changes would aid in the precise application of disease-modifying therapies for the most susceptible individuals, as well as comprehensive monitoring of the treatment response. These would jointly contribute to the development of efficient disease-modifying treatments, which cleverly target AD pathophysiology in the appropriate patients at the correct stage of disease.

## Supplementary information

Supplementary table 1, table 2, table 3, table 4
