# Supplementary material for: New fluid biomarkers tracking non-amyloid-β and non-tau pathology in Alzheimer’s disease
Source: Exp Mol Med. 2020 Apr 13;52(4):556–68. doi: 10.1038/s12276-020-0418-9 (PMC7210893; doi:10.1038/s12276-020-0418-9)
Supplement: Supplementary file 1 — Supplementary table 1, table 2, table 3, table 4 [file 12276_2020_418_MOESM1_ESM.doc]

New Fluid Biomarkers Tracking Non-Amyloid-β and Non-Tau Pathology in Alzheimer’s Disease

Sun Ah Park^1,2,3^, Song Mi Han^1,3^, Chae Eun Kim^1,3^

**SUPPLEMENT INFORMATION**

Supplementary Table 1, Table 2, Table 3 and Table 4

**Supplementary table 1. New fluid biomarkers: Neurodegeneration-related biomarkers**

| biomarker, *gene*; function | findings in AD; measurement tool [study group] |
| --- | --- |
| chromogranin-A, *CHGA*; dense core secretory granule | CSF:  ①-1. ↓AD vs. CON; SRM [88 CON, 142 MCI, 65 AD]^1^; SRM [15 CON, 10 AD]^2^, ①-2. →AD vs. CON; SRM [40 CON, 23 MCI-S, 14 MCI-AD, 3 MCI-O, 40 AD]^3^  ② ↑MCI vs. CON^3^  ③ correlation with EC and H volume^1^  ④ correlation with CSF tTau and pTau^1^ |
| contactin-2, *CNTN2*; organizing node of Ranvier, cell adhesion | CSF:  ①-1. ↑AD vs. CON; SRM [23 CON, 20 MCI-AD, 10 ADD]^4^, ①-2. ↓AD vs. CON; ELISA [50 CON, 106 AD]^5^  ②↑MCI vs. CON^4^  ③ correlation with cognitive function (MMSE and CDR)^4^ |
| myelin basic protein, *MBP*; myelin formation and stabilization | CSF:  ① ↑AD vs. CON, ↓AD vs. SVD; ELISA [30 CON, 30 AD, 26 SVD]^6^  ② → NL-Aβ (+) vs. NL-Aβ (-); ELISA [86 NL-Aβ (-), 43 NL-Aβ (+)]^7^ |
| neurofascin, *NFASC*; neurite outgrowth, synaptic stabilization | CSF:  ① ↓AD vs CON; SRM [15 CON, 10 AD]^2^  ② ↑MCI vs. CON; SRM [40 CON, 23 MCI-S, 14 MCI-AD, 3 MCI-O, 40 ADD]^3^ |
| neurofilament light polypeptide, *NEFL*; maintenance of neuronal caliber | CSF:  ① ↑AD vs. CON; ELISA [30 CON, 30 AD, 26 SVD]^6^; ELISA [109 CON, 187 MCI (65 MCI-S, 104 MCI-C, with 2 yr FU), 93AD]^8^; ELISA [110 CON, 101 MCI-AD, 91 MCI-S, 95 AD]^9^  ② ↑MCI vs. CON^9^  ③-1. ↑NL-Aβ (+)/MCI-Aβ (all)/AD-Aβ (all) vs. NL-Aβ (-); Simoa immunoassay [57 NL-Aβ (-), 37 NL-Aβ (+), 36 MCI-Aβ (-), 109 MCI-Aβ (+), 5 AD-Aβ (-), 65 AD-Aβ (+)]^10^ ③-2. ↑ADD (+) vs. CON (–)/CON (+)/MCI (+); ELISA [31 CON (-), 13 CON (+), 13 MCI (-), 23 MCI (+), 32 ADD (+), 13 Non-ADD]^11^ ③-3. ↑AD-Aβ (-) > MCI-Aβ (-) > CON-Aβ (-), ↑AD-Aβ (+) vs. CON-Aβ (+)/MCI-Aβ (+), ↑MCI-Aβ (+) vs. MCI-Aβ (-), ↑CON-Aβ (+) vs. CON-Aβ (-); immunoassay [95 CON-Aβ (-), 45 CON-Aβ (+), 187 MCI-Aβ (-), 263 MCI-Aβ (+), 23 AD-Aβ (-), 157 AD-Aβ (+), with 2.3 yr FU (mean)]^12^  ④ ↑AD-DS vs. asymptomatic DS; ELISA [67 CON, 194 asymptomatic DS, 39 prodromal AD-DS, 49 AD-DS]^13^  ⑤ → MCI-S vs. MCI-C; ELISA [109 CON, 187 MCI (65 MCI-S, 104 MCI-C, with 2 yr FU), 93 AD]^8^; ELISA [552 MCI-S, 96 MCI-C, with > 1 yr FU) ^14^  ⑥-1. correlation with MMSE and ADAS-cog in AD^9^ ⑥-2. association with cognitive decline in CON-Aβ (+)^12^ ⑥-3. association with cognitive decline in MCI-Aβ (all); ELISA [109 CON, 187 MCI (65 MCI-S, 104 MCI-C, with 2 yr FU), 93 AD]^8^ or immunoassay^12^ ⑥-4. association with cognitive decline in NL with AD risk; ELISA [150 NL]^15^  ⑦-1. association with faster brain atrophy^9^ ⑦-2. association with brain atrophy in all; Simoa immunoassay [57 NL-Aβ (-), 37 NL-Aβ (+), 36 MCI-Aβ (-), 109 MCI-Aβ (+), 5 AD-Aβ (-), 65 AD-Aβ (+)]^10^  ⑧-1. correlation with CSF Aβ_42_ levels; ELISA^9^ or Simoa immunoassay^10^, ⑧-2. no correlation with CSF Aβ_42_ levels; ELISA^11^  ⑨ correlation with CSF tTau and pTau_181_ levels^9,10,11^  Blood:  ① ↑MCI/AD vs. CON (AUC on ROC, 0.87); Simoa immunoassay [193 CON, 197 MCI, 180 AD, with 2 yr FU]^16^; ELISA [1583 subjects in ADNI; 401 CON, 855 MCI, 327 AD with annual collection for < 11 yr]^17^  ②-1. ↑MCI-Aβ (+) vs. NL-Aβ (-), ↑AD-Aβ (+) vs. NL-Aβ (-); Simoa immunoassay [57 NL-Aβ (-), 37 NL-Aβ (+), 36 MCI-Aβ (-), 109 MCI-Aβ (+), 5 AD-Aβ (-), 65 AD-Aβ (+)]^10^, ②-2.↑MCI/AD vs. CON^17^, ②-3. ↑presymptomatic stages of fAD with mutation vs. without mutation; Simoa immunoassay [405 DIAN subjects]^18^  ③ ↑prodromal AD-DS vs. asymptomatic DS, ↑AD-DS vs. asymptomatic DS; Simoa immunoassay [67 CON, 194 asymptomatic DS, 39 prodromal AD-DS, 49 ADD-DS]^13^  ④-1. ↑PSP vs. CON; immunoassay [12 CON, 162 PSP]^19^, ④-2. ↑atypical PD (MSA, PSP, and CBD) vs. typical PD/CON; Simoa immunoassay [79 CON, 244 PD, 88 MSA, 70 PSP, 23 CBD]^20^  ⑤-1. association with cognitive decline^16,17^, ⑤-2. association with severe neurological, functional, and neuropsychological decline in PSP with 1 yr FU^19^, ⑤-3. peaked increase at conversion from the presymptomatic to the symptomatic stage in fAD^18^  ⑥-1. association with rapid brain regional atrophy^16,17,18,21^ ⑥-2. association with greater brain atrophy in PSP with 1 yr FU^19^, ⑥-3. association with greater brain hypometabolism^16,17^  ⑦-1. correlation of blood NFL with CSF NFL levels^10,13,16,18,19,20,22^, ⑦-2. association of longitudinal ↑NFL level in CSF AD biomarker (+)^17^ |
| neurogranin, *NRGN*; calmodulin-binding postsynaptic neuronal protein | CSF:  ① ↑AD vs, CON; immunoassay [37 CON, 61 MCI, 65 AD, with 2 yr FU-LP]^23^; ELISA [80 CON, 40 MCI, 100 AD]^24^; hybrid immunoaffinity-MS [37 CON, 38 AD]^25^; Immunoassay [110 CON, 173 MCI, 95 AD, with 2 yr FU]^26^; ELISA [53 CON, 62 MCI-S, 35 MCI-C, 74 AD, 47 LBD/PDD, 34 VD, 33 FTD]^27^; immunoassay [207 CON, 95 AD]^28^; ELISA [19 CON, 100 AD, 2 ADAD, 20 bvFTD, 21 svFTD, 13 LBD, 31 PD, 46 PSP, 29 MSA]^29^  ② ↑MCI-AD vs, CON^26^  ③-1. ↑MCI-Aβ (+)/AD-Aβ (+) vs. NL-Aβ (-); immunoassay [57 NL-Aβ (-), 37 NL-Aβ (+), 36 MCI-Aβ (-), 109 MCI-Aβ (+), 5 AD-Aβ (-), 65 AD-Aβ (+)]^10^, ③-2. ↑AD-Aβ (+) vs. MCI-Aβ (-)/CON- Aβ (all), ↑MCI-Aβ (+) vs. MCI-Aβ (-)/CON-Aβ (-); ELISA [35 CON-Aβ (-), 21 CON-Aβ (+), 18 MCI-Aβ (-), 58 MCI-Aβ (+), 16 AD-Aβ (+), with 1-7 yr FU-LP]^30^, ③-3. ↑CON-Aβ (+) vs CON-Aβ (-), ↑MCI-Aβ (+) vs MCI-Aβ (-), ↑ADD-Aβ (+) vs ADD-Aβ (-)^12^, ③-4. ↑NL-Aβ (+) vs. NL-Aβ (-); ELISA [86 NL-Aβ (-), 43 NL-Aβ (+)]^7^  ④-1. ↑AD vs. OD; ELISA [13 MCI-AD, 29 MCI-O, 14 non-ADD, 39 AD]^31^; ELISA [53 CON, 62 MCI-S, 35 MCI-C, 74 AD, 47 PD/LBD, 34 VD, 33 FTD]^27^; ELISA [19 CON, 100 AD, 2 ADAD, 20 bvFTD, 21 svFTD, 13LBD, 31 PD, 46 PSP, 29 MSA]^29^; ELISA [75 CON, 114 MCI, 397 AD, 6 PCA, 96 FTD, 68 ALS, 37 PD, 19 MCI-PD, 29 PDD, 33 LBD, 21 CBD, 20 PSP]^32^, ④-2. ↑MCI-AD vs. MCI-O^31^  ⑤ ↑MCI-C vs. MCI-S; immunoassay [37 CON, 61 MCI, 65 AD, with 2 yr FU-LP]^23^; Immunoassay [109 CON, 65 MCI-S, 104 MCI-C, with 2 yr FU, 93 AD]^8^; ELISA^24^; Immunoassay^26^  ⑥-1. continual decrease in AD-Aβ (+) on 1-7 yr FU-LP^30^, ⑥-2. association of decreased levels with cognitive decline in MCI-Aβ (+)^12^, ⑥-3 association of increased levels with cognitive decline in ADD-Aβ (+)^26^; ELISA [109 CON, 187 MCI (65 MCI-S, 104 MCI-C, with 2 yr FU), 93 AD]^8^; immnoassay^12^  ⑦-1. association with regional brain atrophy in CON (NL)-Aβ (+); immunoassay [207 CON, 95 AD]^28^; immunoassay [57 NL-Aβ (-), 37 NL-Aβ (+), 36 MCI-Aβ (-), 109 MCI-Aβ (+), 5 AD-Aβ (-), 65 AD-Aβ (+)]^10^; immunoassay [16 NL-Aβ (+), 22 NL-Aβ (-)]^33^; ELISA^8^, ⑦-2. association with H atrophy and longitudinal reductions in cortical glucose metabolism in MCI^26^  ⑧-1. correlation with CSF Aβ_42_; ELISA [53 CON, 62 MCI-S, 35 MCI-C, 74 ADD, 47 PDD/LBD, 34 VD, 33 FTD]^27^; ELISA^8,32^; Simoa immunoassay^10^, ⑧-2. correlation with Aβ load in preclinical AD; immunoassay [207 CON, 95 AD]^28^, ⑧-3. no correlation with CSF Aβ_42_^23,31^  ⑨ correlation with CSF tTau and/or pTau; ELISA [29 CON, 59 MCI-AD]^34^; ELISA^31^; immunoassay^23^; SRM [48 CON, 72 MCI or mild AD]^35^  Blood:  ①-1. ↓AD/FTD vs. CON in NDE; ELISA [28 CON, 12 AD, 16 FTD (19 CON, 9 AD, 10 FTD, with FU)]^36^, ①-2. ↓AD vs. CON in NDE; ELISA using NDE [10 CON, 10 AD, 20 MCI-S, 20 MCI-C, with 3 yr FU]^37^, ①-3. →AD vs. CON; ELISA [29 CON, 59 MCI-AD]^34^; meso scale discovery immunoassay and hybrid immunoaffinity-MS [37 CON, 38 AD]^25^  ② ↓MCI-C vs. MCI-S in NDE^37^  ③ no correlation between CSF and plasma neurogranin^25^ |
| neuronal pentraxin 1, *NPTX1*; synapse formation & stabilization | CSF:  ① ↓AD vs. CON; SRM [15 CON, 10 AD]^2^; SRM [40 CON, 3 MCI-S, 14 MCI-AD, 3 MCI-O, 40 ADD]^3^  ② ↑MCI vs. CON^3^ |
| secretogranin-2, *SCG2*; dense core secretory granule proteins | CSF:  ① ↓AD vs. CON; SRM [15 CON, 10 AD]^2,3^  ② ↑MCI vs. CON; SRM [40 CON, 3 MCI-S, 14 MCI-AD, 3 MCI-O, 40 ADD]^3^  ③ ↓MCI-C vs. MCI-S; SRM [85 CON, 67 MCI-S, 67 MCI-C, MCI, 66 AD]^38^ |
| synaptosomal-associated protein 25, *SNAP25*; neurotransmitter release | CSF:  ①-1. ↑AD vs. CON; SRM [15 CON, 10 AD]^2^; ①-2. ↑AD vs. CON/MCI-Aβ (-); ELISA [35 CON-Aβ (-), 21 CON-Aβ (+), 18 MCI-Aβ (-), 58 MCI-Aβ (+), 16 AD-Aβ (+), with 1-7 yr FU-LP]^30^  ② ↑MCI-Aβ (+) vs. CON/MCI-Aβ (-)^30^  ③ Longitudinally decrease in AD on 1-7 yr FU-LP^30^ |
| visinin-like protein 1, *VSNL1*; neuronal calcium sensor protein | CSF:  ①-1. ↑AD vs. CON; microparticle based immunoassay [211 CON, 98 AD, 11 FTD, 7 PSP, 1 LBD]^39^; microparticle based immunoassay [211 CON, 60 AD, with 2.6 yr FU]^40^; ELISA [40 CON, 61 AD, 32 LBD]^41^; ELISA [18 CON, 15 MCI, 33 AD]^42^; microparticle based immunoassay [64 CON, 23 AD, with 2-3 yr FU]^43^; ELISA [9 CON, 45 MCI, 109 AD, 9 VD, 18 FTD, 5 LBD]^44^; ELISA [35 CON-Aβ (-), 21 CON-Aβ (+), 18 MCI-Aβ (-), 58 MCI-Aβ (+), 16 AD-Aβ (+), with 1-7 yr FU-LP]^30^, ①-2. →AD vs. CON; ELISA [37 CON, 61 MCI, 65 AD]^45^  ②-1. ↑MCI-Aβ (+) vs. CON-Aβ (-)^30^, ②-2. →MCI vs. CON; ELISA [37 CON, 61 MCI, 65 AD]^45^, ②-3. ↑AD vs. MCI^42^  ③-1. ↑AD vs. LBD^41^, ③-2. ↑AD vs. OD^39^, ③-3. → AD vs. VD, →AD vs. FTD^44^  ④ ↑MCI-C vs. MCI-S^45^  ⑤ longitudinal decrease in AD-Aβ (+)^30^  ⑥ association of VILIP-1 and VILIP-1/Aβ_42_ with future cognitive impairment and its speed in MCI, pre-MCI state, and CON^39^  ⑦-1. correlation with brain regional volume in NL; immunoassay [16 NL-Aβ (+) vs. 22 NL-Aβ (-)]^33^, ⑦-2. association with rate of brain regional atrophy^43^  ⑧-1. correlation with CSF tTau and/or pTau_181_ levels^39^, ⑧-2. correlation with CSF Aβ_42_/Aβ_40_ ratio^42^  Blood:  ① ↑AD vs. CON; microparticle based immunoassay [211 CON, 98 AD, 11 FTD, 7 PSP, 1 LBD]^39^ |

*The italics are the gene names of individual protein biomarkers*

AD, Alzheimer’s disease; ADAD, autosomal-dominant AD; ADAS-cog, AD assessment scale–cognitive subscale; ADD, AD dementia; AD-DS, down syndrome with AD; ADNI, Alzheimer’s disease neuroimaging initiative; ALS, amyotrophic lateral sclerosis; α-syn, alpha-synuclein; A/T/N, amyloid pathology-related biomarker / tau pathology-related biomarker / neurodegeneration-related biomarker^46^; AUC, area under the curve; bvFTD, behavioral variant frontotemporal dementia; CBD, corticobasal degeneration; CDR, clinical dementia rating; CON, control; CSF, cerebrospinal fluid; DIAN, the dominantly inherited Alzheimer network; DS, down syndrome; EC, entorhinal cortex; ELISA, enzyme-linked immunosorbent assay; fAD, familial AD; FTD, frontotemporal dementia; FU, follow-up; H, hippocampus; LBD, Lewy body dementia; LP, lumbar puncture; MCI-AD, mild cognitive impairment due to AD; MCI-C, MCI with conversion to AD on follow-up; MCI-O, MCI due to other neurodegenerative disorder (non-AD); MCI-PD, MCI due to PD; MCI-S, MCI with no cognitive decline on follow-up; MMSE, mini-mental state examination; MS, mass spectrometry; MSA, multisystem atrophy; NFL, neurofilament-light chain; NDE, neuron-derived exosome; NL, cognitively normal; OD, other disease; PD, Parkinson’s disease; PDD, PD with dementia; PSP, progressive supranuclear palsy; ROC, receiver operating characteristic curve; SRM, selected reaction monitoring; svFTD, speech variant FTD; SVD, subcortical vascular dementia; tTau, total tau protein; pTau, phosphorylated tau protein; VD, vascular dementia; VILIP-1, visinin-like protein 1; yr, year; 2D, two-dimension

(+)/(-) , positive or negative AD biomarker, either CSF or amyloid positron emission tomography (PET)

↑, increased protein level; ↓, decreased protein level; →, no change in protein level

**Supplementary table 2. New fluid biomarkers: I**nflammation-related biomarkers

| biomarker, *gene*; function | findings in AD; measurement tool [study group] |
| --- | --- |
| β2-microglobulin, *B2M*; antigen presentation to the immune system | CSF:  ① ↑MCI vs. CON; SRM [40 CON, 3 MCI-S, 14 MCI-AD, 3 MCI-O, 40 ADD]^3^  ② ↑MCI-C vs. MCI-S; protein chip array followed by MS [28 CON, 56 MCI-S, 57 MCI-AD, with 4-6 yr FU]^47^ |
| intercellular adhesion molecule 1, *ICAM1*; ligands for the leukocyte adhesion protein, leukocyte trans-endothelial migration | CSF:  ① ↑preclinical, prodromal, and dementia stages of AD vs. CON; meso scale discovery immunoassay [508 CON, 256 MCI, 57 ADD]^48^  ② ↑AD-Aβ (+) vs. CON, ↑AD-Aβ (+) vs. NL-Aβ (+); immunoassay [36 CON, 19 NL-Aβ (+), 39 MCI-Aβ (+), 27 AD-Aβ (+)]^49^  ③ ↑MCI-Aβ (+) vs. CON, ↑MCI-Aβ (+) vs. NL-Aβ (+)^49^  ④-1. ↑MCI-C vs. MCI-S, association with speed of cognitive decline in MCI-C; meso scale discovery immunoassay [30 CON, 52 MCI-S, 47 MCI-C to AD, 20 MCI-C to OD, with 5 yr FU]^50^, ④-2. correlation with cognitive dysfunction (CDR-SOB, MMSE)^48^  ⑤ correlation with CSF pTau^48^ |
| progranulin, *GRN*: regulation of lysosomal function and inflammation as a growth factor | CSF:  ①-1. ↑MC vs. NC; ELISA [130 MC and 85 NC from DIAN cohort; 128 CON, 56 preclinical AD with A(+)/TN(-), 48 preclinical AD with A(+)/TN(+), 289 AD with CDR 0.5, 81 AD with CDR 1 , 182 SNAP from ADNI cohort]^51^, ①-2. ↑AD with CDR 1 vs. CON, ↑SNAP > AD with CDR 1 > AD with CDR 0.5 > preclinical AD with A(+)/TN(-), ↑preclinical AD with A(+)/TN(+) vs. preclinical AD with A(+)/TN(−)^51^  ②-1. association with cognitive scores in AD^51^. ②-2. association with decreased temporo‐parietal FDG‐PET uptake in AD^51^, ②-3. no association with total H volume^51^  ③-1. correlation with tTau and pTau in AD, but not in CON^51^, ③-2. correlation with sTREM2 in AD, but not in CON^51^ |
| osteopontin, *SPP1*: secreted glyco-phosphoprotein with a role in cell-matrix interaction and innate immunity | CSF:  ①-1. ↑AD vs. CON; ELISA [69 CON, 67 AD, 46 FTD]^52^; MS followed by SRM [30 CON, 27 LBD, 16 AD, 7 PD]^53^; SRM [10 ADD, 23 CON, 20 MCI-AD]^4^, ①-2. ↑AD-newly diagnosed vs. AD-chronic, ↑AD-newly diagnosed vs. MCI, ↑AD-newly diagnosed vs. CON, ↑AD-newly diagnosed vs. OD; ELISA [20 CON, 18 MCI-S, 13 MCI-C, 17 AD-newly diagnosed, 18 AD-chronic, 20 OD]^54^  ② ↑MCI-AD vs. CON^4^  ③-1. ↑AD vs. FTD; ELISA [69 CON, 67 AD, 46 FTD]^52^; ③-2. ↑AD-newly diagnosed vs. OD^54^, ③-3. ↑AD vs. non-AD; SRM [81 AD, 67 non-AD (FTD, LBD, healthy CON)]^55^, ③-4. ↑AD/LBD/PD vs. CON; MS followed by SRM [30 CON, 27 LBD, 16 AD, 7 PD]^53^, ③-5. ↑AD vs PD, ↓PD vs. CON; SRM [70 CON, 38 AD, 70 PD]^56^, ③-6. ↑multiple sclerosis vs. OD; ELISA [24 CON, 44 OD, 103 multiple sclerosis]^57^  ④↑MCI-C vs. MCI-S; MS [56 MCI-S, 57 MCI-C, 28 CON, with 4-6 yr FU]^47^  ⑤ correlation with cognitive decline (MMSE)^52^  ⑥ correlation with tTau and pTau^55^ |
| soluble triggering receptor expressed on myeloid cells 2 (sTREM2), *TREM2;* transmembrane protein expressed on the surface of microglia involved in innate immunity | CSF:  ①-1. ↑AD vs. CON; MS followed by SRM [30 CON, 16 AD, 27 LBD, 7 PD]^53^; SRM [22 CON, 37 AD]^58^; ELISA [107 CON, 73 AD, 40 TREM2 risk gene variants]^59^; ELISA [85 CON, 130 MCI, 116 AD]^60^; SRM [15 CON, 13 AD, 32 FTD]^61^, ①-2. ↑AD at CDR 0.5 vs. CON, ↑NL-A(+)/TN(+) vs. CON, ↑NL-A(+)/TN(+) vs. NL-A(+)/TN(-), ↓NL-A(+)/TN(-) vs. CON; ELISA [122 CON, 52 NL-A(+)/TN(-), 45 NL-A(+)/TN(+), 282 AD with CDR 0.5, 80 AD with CDR 1 from ADNI]^62^, ①-3. → CON vs. MCI vs. AD; ELISA [75 CON, 21 MCI, 54 AD]^63^; ELISA [49 CON, 19 preclinical AD, 27 MCI-AD, 15 mild AD]^64^, ①-4. ↑p.R47H variant in TREM2 gene vs. NC, ↓p.L211P variant vs. NC; ELISA [1027 from ADNI cohort (984 NC, 20 p.R62H, 7 p.R47H, 11 p.L211P, 3 p.D87N, 1 p.R62H/p.D87N, p.H157Y)]^62^  ②-1. ↑MCI vs. CON^60^, ②-2. ↑MCI-AD vs. CON, ↑MCI-AD vs. preclinical AD, ↑MCI-AD vs. AD; ELISA [150 CON, 63 NL-Aβ (+), 111 MCI-Aβ (+), 200 AD]^65^, ②-3.↑MC with mild AD vs. NC; ELISA [91 NC, 127 MC from DIAN cohort]^66^  ③-1. ↑NL-A(-)/TN(+) (cognitively normal subjects with suspected non-AD pathology) vs. CON, ↑SNAP (NL-A(-)/TN(+)) vs. preclinical AD (NL-A(+)/TN(-))^65^, ③-2. ↑NL-Aβ (+)/MCI-Aβ (+)/AD vs. CON, ↑MCI-Aβ (+) vs. AD/NL-Aβ (+); ELISA [36 CON, 19 NL-Aβ (+), 39 MCI-Aβ (+), 27 AD]^49^  ④-1. ↑LBD vs. CON^53^, ④-2. →AD vs. FTD, ↑FTD vs. CON; SRM [15 CON, 13 AD, 32 FTD]^61^  ⑤ no association with cognitive change; ELISA [138 CON, 302 MCI, 57 ADD]^67^  ⑥-1. correlation with CSF tTau and pTau^65^, ⑥-2. no correlation with CSF Aβ_42_^67^, ⑥-3. correlation with CSF Aβ_42_^63^  ⑦-1. correlation with age in all group^65^, ⑦-2. correlation with age in CON^63^  Blood:  ① → CON vs. AD^59^  ② ↑NL-AD/NL-VD vs. NL-S; ELISA [1,349 NL ≥ 60 yr-old, with 10 yr FU (300 NL-D (193 NL-AD*, 85 NL-VD, 22 NL-OD), 1049 NL-S)]^68^  ③ no correlation between CSF and plasma sTREM2 levels^59^  ④ no correlation with CSF tTau, pTau_181_ and Aβ_42_^59^ |
| chitinase-3-like protein 1 (YKL-40), *CHI3L1*:  secreted glycoprotein from reactive astrocyte and microglia with a modulating role of inflammation | CSF:  ① ↑AD vs. CON; ELISA [198 CON, 65 AD with CDR 0.5, 29 AD with CDR 1]^69^; ELISA [19 CON, 13 MCI-S, 25 AD, 11 OD]^70^; SRM [10 CON, 5 MCI, 45 AD]^71^; ELISA [53 CON, 62 MCI-S, 35 MCI-C, 74 ADD, 47 PDD/LBD, 34 VD, 33 FTD]^27^; SRM [81 AD, 67 non-AD (FTD, LBD, NL)]^55^; ELISA [508 CON, 256 MCI, 57 AD]^48^; immunoassay [35 CON-Aβ (-), 21 CON- Aβ (+), 18 MCI- Aβ (-), 58 MCI- Aβ (+), 16 AD, with 1-7 yr FU-LP]^30^; immunoassay [36 CON, 19 NL-Aβ (+), 39 MCI-Aβ (+), 27 AD]^49^  ②-1. ↑AD vs. MCI-Aβ (+)^49^, ②-2. ↑preclinical AD/MCI/AD vs. CON; ELISA [49 CON, 19 preclinical AD, 27 MCI-AD, 15 mild AD]^64^  ③-1. ↑NL-A(all)/TN(+) vs. ↑NL-A(all)/TN(-); ELISA [203 NL-A(-)/T(-)/N(-), 26 NL-A(+)/TN(-), 10 NL-A(+)/TN(either +), 27 NL-A(-)/TN(either+)]^72^, ③-2. ↑CON-Aβ (+) vs CON-Aβ (-), ↑MCI-Aβ (+) vs. MCI-Aβ (-), → AD-Aβ (-) vs. 157 AD-Aβ (+); ELISA [95 CON-Aβ (-), 45 CON-Aβ (+), 187 MCI-Aβ (-), 263 MCI-Aβ (+), 23 AD-Aβ (-), 157 AD-Aβ (+), with 2.3 yr FU (mean)]^12^, ③-3. → NL-Aβ (-) vs. NL-Aβ (+); ELISA [86 NL-Aβ (-), 43 NL- Aβ (+)]^7^  ④-1. ↑AD/LBD vs. CON; SRM [30 CON, 27 LBD, 16 AD, 7 PD]^53^, ④-2. ↑AD vs. CON/MCI-S/LBD/PD^27^, ④-3.↑AD vs. no-AD^55^, ④-4. ↑FTD/AD vs. CON; ELISA [77 CON, 97 AD, 49 FTD]^73^, ④-5. ↑VD/ FTD, vs. CON^27^, ④-6. ↑AD vs. LBD/PD/CON; ELISA [44 CON, 49 AD, 61 PD, 36 LBD]^74^  ⑤-1. ↑MCI-C vs. MCI-S; ELISA [37 CON, 61 MCI, 65 AD]^30^, ⑤-2. → MCI-C vs. MCI-S^27^, ⑤-3. longitudinally increase over time in MCI, not in AD^30^, ⑤-4. association with advanced clinical stage in the absence of Aβ pathology (CON-Aβ (-) < MCI-Aβ (-) < ↑ADD-Aβ (-)^12^, ⑤-4. ↓CON- Aβ (+)/MCI Aβ (+) vs. AD Aβ (+)^12^  ⑥-1. correlation with cognitive decline (MMSE, CDR-SOB)^48^, ⑥-2. no associations with MMSE; ELISA [13 MCI-AD, 39 ADD, 29 MCI-O, 14 non-ADD]^31^, ⑥-3. correlation with age^72^, ⑥-4. correlation with gray matter atrophy; ELISA [53 CON, 20 NL-Aβ (+), 28 MCI-AD, 15 mild AD]^75^; ELISA^75^; immunoassay [16 NL-Aβ (+) vs. 22 NL-Aβ (-)]^33^  ⑦-1. correlation with CSF tTau & pTau_181_; ELISA^48^; SRM^55^, ⑦-2. correlation with tTau and pTau in non-AD group, no correlation with tTau and pTau in AD group; ELISA [13 MCI-AD, 39 ADD, 29 MCI-O, 14 non-ADD]^31^, ⑦-3. no association with CSF pTau & tTau^74^, ⑦-4. correlation with pathological tau burden on autopsy^73^  ⑧ no correlation with Aβ^74^  Blood:  ① ↑AD vs. CON; ELISA^69^; ELISA [35 CON, 49 MCI, 41 AD-mild, 20 AD-severe]^76^  ② ↑CJD vs. CON, ↑LBD vs. CON; ELISA [70 CON, 50 AD, 17 FTD, 78 CJD, 34 LBD, 22 VD, 44 OD]^77^  ③-1. correlation with dementia severity (CDR and NPI)^76^ ③-2. correlation with age^69^  ④ no correlation with CSF Aβ_42_, tTau, pTau and cortical amyloid load^69^  ⑤ modest correlation between plasma and CSF YKL-40 levels (roughly 5-fold higher in CSF)^69^ |

*The italics are the gene names of individual protein biomarkers*

AD, Alzheimer’s disease; ADD, Alzheimer’s disease dementia; ADNI, Alzheimer’s disease neuroimaging initiative; A/T/N, amyloid pathology-related biomarker / tau pathology-related biomarker / neurodegeneration -related biomarker^46^; CDR, clinical dementia rating; CDR-SOB, CDR-sum of box; CJD, Creutzfeldt-Jakob disease; CON, control; CSF, cerebrospinal fluid; D, dementia; DIAN, the dominantly inherited Alzheimer network; ELISA, enzyme-linked immunosorbent assay; FDG-PET, fluorine-18 fluoro-deoxy-glucgose-ositron emission tomography; FTD, frontotemporal dementia; FU, follow-up; H, hippocampus; LBD, Lewy body dementia; MC, mutation carriers; MCI, mild cognitive impairment; MCI-AD, MCI due to AD; MCI-C, MCI with conversion to AD on follow-up; MCI-O, MCI due to other neurodegenerative disorder; MCI-S, MCI with no cognitive decline on follow-up; MMSE, mini-mental state examination; MS, mass spectrometry; NC, non-carrier; NL, cognitively normal; NL-AD, cognitively normal subjects with future development of AD on follow-up; NL-OD, cognitively normal subjects with future development of OD on follow-up; NL-S, cognitively normal subjects without dementia on follow-up; NL-VD, cognitively normal subjects with future development of VD on follow-up; NPI, neuropsychiatric inventory; OD, other disease; PD, Parkinson’s disease; SMC, subjective memory complaints; SNAP, suspected non-Alzheimer’s pathophysiology; SRM, selected reaction monitoring; TREM2, triggering receptor expressed on myeloid cells 2; tTau, total tau protein; pTau, phosphorylated tau protein; VD, vascular dementia; yr, year

(+)/(-) , positive or negative AD biomarker, either CSF or amyloid PET

↑, increased protein level; ↓, decreased protein level; →, no change in protein level

*the count is overlapped when double pathology was identified

**Supplementary table 3. New fluid biomarkers: Lipid metabolism-related biomarkers**

| biomarker, *gene*; function | findings in AD; measurement tool [study group] |
| --- | --- |
| apolipoprotein E, *APOE*; regulation of lipid homeostasis and redistribute cholesterol and other lipids to neurons | CSF:  ①-1. ↓AD vs. CON; immunoassay [92 CON, 149 MCI, 69 AD]^78^, ①-2. ↑AD vs. CON; MS followed by SRM [30 CON, 27 LBD, 16 AD, 7 PD]^53^  ② ↓AD vs. MCI^78^  ③-1. ↑AD vs. LBD, ↑LBD vs. CON^53^, ③-2. ↑AD vs. OD (non-AD); SRM [81 AD, 67 non-AD (FTD, LBD, healthy CON)^55^; immunoassay [18 CON, 13 MCI-S, 29 AD, 14 OD]^79^, ③-3. ↓OD vs. CON^79^  ④ association of low ApoE with cognitive decline, MCI conversion to dementia, and gray matter atrophy rate in *APOE*ε4 (-)^78^  ⑤-1. correlation with tTau and pTau^79^, ⑤-2. no correlation with Aβ_42_^78^, ⑤-3. ↓ApoE4 carriers vs. ApoE2 carriers, ↓ApoE3 carriers vs. ApoE2 carriers; immunoassay [88 CON, 142 MCI, 65 AD]^1^, ⑤-4. → ApoE2 vs. E3 vs. E4 carriers^79^ |
| fatty acid binding protein, heart, *FABP3*; fatty acid metabolism and lipid transport | CSF:  ① ↑AD vs. CON; ELISA [30 CON, 30 AD, 26 VD]^6^; immunoassay [90 CON, 139 MCI, 66 AD]^80^; immunoassay [92 CON, 149 MCI, 69 AD]^81^; ELISA [65 CON, 170 MCI, with 5.7 yr FU (mean), 96 AD]^82^; immunoassay [88 CON, 142 MCI, 65 AD]^1^; immunoassay [325 CON, 344 AD]^83^; ELISA [31 CON(-), 13 CON (+), 13 MCI (-), 23 MCI (+), 32 ADD (+), 13 Non-ADD (-)]^11^  ② ↑MCI vs. CON^11^  ③-1. ↑VD vs. CON^6^, ③-2. ↑AD vs. PD only, ↑AD vs. OD, ↑LBD vs. PD only, →PD only vs. OD; ELISA [48 AD, 40 LBD, 20 PDD, 54 PD only, 46 OD]^84^  ④ ↑MCI-C vs. MCI-S^81^  ⑤ correlation with MMSE^84^  ⑥ correlation with longitudinal brain regional atrophy in all group-Aβ (+)^80^  ⑦ correlation with tTau and pTau^84^  ⑧-1. no correlation with CSF Aβ_42_^84^, ⑧-2. ↑NL-Aβ (+) vs. NL-Aβ (-); immunoassay [86 NL-Aβ (-), 43 NL-Aβ (+)]^7^ |

*The italics are the gene names of individual protein biomarkers*

AD, Alzheimer’s disease; ADD, Alzheimer’s disease dementia; ApoE, apolipoprotein E; CON, control; CSF, cerebrospinal fluid; D, dementia; ELISA, enzyme-linked immunosorbent assay; FTD, frontotemporal dementia; FU, follow-up; LBD, Lewy body dementia; MCI, mild cognitive impairment; MCI-C, MCI with conversion into AD on follow-up; MCI-S, MCI with no cognitive decline on follow-up; MMSE, mini-mental state examination; MS, mass spectrometry; NL, cognitively normal; OD, other disease; PD, Parkinson’s disease; SRM, selected reaction monitoring; tTau, total tau protein; pTau, phosphorylated tau protein; VD, vascular dementia; yr, year

(+)/(-) , positive or negative AD biomarker, either CSF or amyloid PET

↑, increased protein level; ↓, decreased protein level; →, no change in protein level

**Supplementary table 4. New fluid biomarkers: biomarkers related to protein clearance**

| biomarker, *gene*; function | findings in AD; measurement tool [study group] |
| --- | --- |
| clusterin, *CLU*; secretory glycoprotein with a role in inhibition of protein aggregation and possible neuroprotection | CSF:  ① ↑AD/LBD vs. CON; MS and SRM [30 CON, 16 AD, 27 LBD, 7 PD]^53^  ② correlation with cognitive decline; immunoassay [629 AD, 581 CON]^85^  ③ correlation with CSF tTau/Aβ_42_ ratio^85^  Blood:  ① → AD vs. CON; immunoassay [831 AD, 524 CON]^85^; immunoassay [452 CON, 169 MCI-S, 51 MCI-C, 476 AD]^86^  ② association with cognitive function (MMSE)^86^  ③ association of increased plasma clusterin with brain regional atrophy^86^  ④ no association with CSF tTau/Aβ_42_ ratio^85^ |
| orexin (hypocretin), *HCRT*; regulation of circadian rhythm by increasing arousal levels | CSF:  ①-1. ↑AD vs. CON; SRM [15 CON, 13 AD, 32 FTD]^61^, ①-2. → AD vs. CON; immunoassay [33 CON, 33 AD]^87^  ② ↑MCI-AD vs. CON; RIA [24 CON, 41 MCI-AD, 41 AD, 24 OD]^88^  ③-1. ↑AD vs. FTD, ↑FTD vs. CON^61^, ③-2. ↑AD/MCI-AD vs. OD^88^, ④-1. correlation with CSF Aβ_42_^88^, ④-2. correlation with pTau_181_ in NL; RIA [63 NL]^89^ |
| transthyretin, *TTR*; binding to Aβ proteins and inhibition of Aβ aggregations | CSF:  ①-1. ↑AD vs. CON; MS followed by SRM [30 CON, 27 LBD, 16 AD, 7 PD]^53^ ①-2. →AD vs. CON; immunoassay [13 CON, 59 AD, 13 LBD]^90^  ②-1. ↑AD vs. LBD^53^, ②-2. →AD vs. LBD, →LBD vs. CON^90^  Blood:  ① ↓serum transthyretin in AD vs. CON; ELISA [90 CON, 111 AD]^91^  ② association of ↓plasma transthyretin with rapid decline in AD on > 6 mon FU; ELISA [270 AD] ^92^  ③ association of ↓plasma transthyretin with severe cognitive impairment in AD^92^ |

*The italics are the gene names of individual protein biomarkers*

AD, Alzheimer’s disease; CON, control; CSF, cerebrospinal fluid; ELISA, enzyme-linked immunosorbent assay; FTD, frontotemporal dementia; LBD, Lewy body dementia; MCI, mild cognitive impairment; MCI-AD, MCI due to AD; MCI-C, MCI with conversion to AD on follow-up; MCI-S, MCI with no cognitive decline on follow-up; MMSE, mini-mental state examination; mon, months; MS, mass spectrometry; OD, other disease; PD, Parkinson’s disease; RIA, radio-immunoassay; SRM, selected reaction monitoring; tTau, total tau protein; pTau, phosphorylated tau protein

(+)/(-), positive or negative AD biomarker, either CSF or amyloid PET

↑, increased protein level; ↓, decreased protein level; → no change in protein level

**References**

1. Khan, W. *et al.* A Subset of Cerebrospinal Fluid Proteins from a Multi-Analyte Panel Associated with Brain Atrophy, Disease Classification and Prediction in Alzheimer's Disease. *PLoS One.* **10**, e0134368 (2015).

2. Brinkmalm, G. *et al.* A Parallel Reaction Monitoring Mass Spectrometric Method for Analysis of Potential CSF Biomarkers for Alzheimer's Disease. *Proteomics Clin. Appl.* **12** (2018).

3. Duits, F. H. *et al.* Synaptic proteins in CSF as potential novel biomarkers for prognosis in prodromal Alzheimer's disease. *Alzheimers Res. Ther.* **10**, 5 (2018).

4. Begcevic, I. *et al.* Brain-related proteins as potential CSF biomarkers of Alzheimer's disease: A targeted mass spectrometry approach. *J. Proteomics.* **182**, 12-20 (2018).

5. Chatterjee, M. *et al.* Contactin-2, a synaptic and axonal protein, is reduced in cerebrospinal fluid and brain tissue in Alzheimer's disease. *Alzheimers Res. Ther.* **10**, 52 (2018).

6. Bjerke, M. *et al.* Cerebrospinal fluid matrix metalloproteinases and tissue inhibitor of metalloproteinases in combination with subcortical and cortical biomarkers in vascular dementia and Alzheimer's disease. *J. Alzheimers Dis.* **27**, 665-676 (2011).

7. Hoglund, K. *et al.* Preclinical amyloid pathology biomarker positivity: effects on tau pathology and neurodegeneration. *Transl. Psychiatry.* **7**, e995 (2017).

8. Mattsson, N. *et al.* Cerebrospinal fluid tau, neurogranin, and neurofilament light in Alzheimer's disease. *EMBO Mol. Med.* **8**, 1184-1196 (2016).

9. Zetterberg, H. *et al.* Association of Cerebrospinal Fluid Neurofilament Light Concentration With Alzheimer Disease Progression. *JAMA Neurol.* **73**, 60-67 (2016).

10. Pereira, J. B., Westman, E. & Hansson, O. Association between cerebrospinal fluid and plasma neurodegeneration biomarkers with brain atrophy in Alzheimer's disease. *Neurobiol. Aging.* **58**, 14-29 (2017).

11. Gangishetti, U. *et al.* Non-beta-amyloid/tau cerebrospinal fluid markers inform staging and progression in Alzheimer's disease. *Alzheimers Res. Ther.* **10**, 98 (2018).

12. Bos, I. *et al.* Cerebrospinal fluid biomarkers of neurodegeneration, synaptic integrity, and astroglial activation across the clinical Alzheimer's disease spectrum. *Alzheimers Dement.* **15**, 644-654 (2019).

13. Fortea, J. *et al.* Plasma and CSF biomarkers for the diagnosis of Alzheimer's disease in adults with Down syndrome: a cross-sectional study. *Lancet Neurol.* **17**, 860-869 (2018).

14. Kern, S. *et al.* Association of Cerebrospinal Fluid Neurofilament Light Protein With Risk of Mild Cognitive Impairment Among Individuals Without Cognitive Impairment. *JAMA Neurol.* **76**, 187-193 (2019).

15. Merluzzi, A. P. *et al.* Differential effects of neurodegeneration biomarkers on subclinical cognitive decline. *Alzheimers Dement (N Y).* **5**, 129-138 (2019).

16. Mattsson, N., Andreasson, U., Zetterberg, H. & Blennow, K. Association of Plasma Neurofilament Light With Neurodegeneration in Patients With Alzheimer Disease. *JAMA Neurol.* **74**, 557-566 (2017).

17. Mattsson, N., Cullen, N. C., Andreasson, U., Zetterberg, H. & Blennow, K. Association Between Longitudinal Plasma Neurofilament Light and Neurodegeneration in Patients With Alzheimer Disease. *JAMA Neurol.* **76**, 791-799 (2019).

18. Preische, O. *et al.* Serum neurofilament dynamics predicts neurodegeneration and clinical progression in presymptomatic Alzheimer's disease. *Nat. Med.* **25**, 277-283 (2019).

19. Rojas, J. C. *et al.* Plasma neurofilament light chain predicts progression in progressive supranuclear palsy. *Ann Clin Transl Neurol.* **3**, 216-225 (2016).

20. Hansson, O. *et al.* Blood-based NfL: A biomarker for differential diagnosis of parkinsonian disorder. *Neurology.* **88**, 930-937 (2017).

21. Rohrer, J. D. *et al.* Serum neurofilament light chain protein is a measure of disease intensity in frontotemporal dementia. *Neurology.* **87**, 1329-1336 (2016).

22. Gisslen, M. *et al.* Plasma Concentration of the Neurofilament Light Protein (NFL) is a Biomarker of CNS Injury in HIV Infection: A Cross-Sectional Study. *EBioMedicine.* **3**, 135-140 (2016).

23. Kester, M. I. *et al.* Neurogranin as a Cerebrospinal Fluid Biomarker for Synaptic Loss in Symptomatic Alzheimer Disease. *JAMA Neurol.* **72**, 1275-1280 (2015).

24. Kvartsberg, H. *et al.* Cerebrospinal fluid levels of the synaptic protein neurogranin correlates with cognitive decline in prodromal Alzheimer's disease. *Alzheimers Dement.* **11**, 1180-1190 (2015).

25. Kvartsberg, H. *et al.* Characterization of the postsynaptic protein neurogranin in paired cerebrospinal fluid and plasma samples from Alzheimer's disease patients and healthy controls. *Alzheimers Res. Ther.* **7**, 40 (2015).

26. Portelius, E. *et al.* Cerebrospinal fluid neurogranin: relation to cognition and neurodegeneration in Alzheimer's disease. *Brain.* **138**, 3373-3385 (2015).

27. Janelidze, S. *et al.* Cerebrospinal fluid neurogranin and YKL-40 as biomarkers of Alzheimer's disease. *Ann. Clin. Transl. Neurol.* **3**, 12-20 (2016).

28. Tarawneh, R. *et al.* Diagnostic and Prognostic Utility of the Synaptic Marker Neurogranin in Alzheimer Disease. *JAMA Neurol.* **73**, 561-571 (2016).

29. Wellington, H. *et al.* Increased CSF neurogranin concentration is specific to Alzheimer disease. *Neurology.* **86**, 829-835 (2016).

30. Sutphen, C. L. *et al.* Longitudinal decreases in multiple cerebrospinal fluid biomarkers of neuronal injury in symptomatic late onset Alzheimer's disease. *Alzheimers Dement.* **14**, 869-879 (2018).

31. Hellwig, K. *et al.* Neurogranin and YKL-40: independent markers of synaptic degeneration and neuroinflammation in Alzheimer's disease. *Alzheimers Res. Ther.* **7**, 74 (2015).

32. Portelius, E. *et al.* Cerebrospinal fluid neurogranin concentration in neurodegeneration: relation to clinical phenotypes and neuropathology. *Acta Neuropathol.* **136**, 363-376 (2018).

33. Schaeverbeke, J. *et al.* Cerebrospinal fluid levels of synaptic and neuronal integrity correlate with gray matter volume and amyloid load in the precuneus of cognitively intact older adults. *J. Neurochem.* **149**, 139-157 (2019).

34. De Vos, A. *et al.* C-terminal neurogranin is increased in cerebrospinal fluid but unchanged in plasma in Alzheimer's disease. *Alzheimers Dement.* **11**, 1461-1469 (2015).

35. Dayon, L. *et al.* Alzheimer disease pathology and the cerebrospinal fluid proteome. *Alzheimers Res. Ther.* **10**, 66 (2018).

36. Goetzl, E. J. *et al.* Decreased synaptic proteins in neuronal exosomes of frontotemporal dementia and Alzheimer's disease. *FASEB J.* **30**, 4141-4148 (2016).

37. Winston, C. N. *et al.* Prediction of conversion from mild cognitive impairment to dementia with neuronally derived blood exosome protein profile. *Alzheimers Dement (Amst).* **3**, 63-72 (2016).

38. Spellman, D. S. *et al.* Development and evaluation of a multiplexed mass spectrometry based assay for measuring candidate peptide biomarkers in Alzheimer's Disease Neuroimaging Initiative (ADNI) CSF. *Proteomics Clin. Appl.* **9**, 715-731 (2015).

39. Tarawneh, R. *et al.* Visinin-like protein-1: diagnostic and prognostic biomarker in Alzheimer disease. *Ann. Neurol.* **70**, 274-285 (2011).

40. Tarawneh, R., Lee, J. M., Ladenson, J. H., Morris, J. C. & Holtzman, D. M. CSF VILIP-1 predicts rates of cognitive decline in early Alzheimer disease. *Neurology.* **78**, 709-719 (2012).

41. Luo, X. *et al.* CSF levels of the neuronal injury biomarker visinin-like protein-1 in Alzheimer's disease and dementia with Lewy bodies. *J. Neurochem.* **127**, 681-690 (2013).

42. Mroczko, B. *et al.* Evaluation of visinin-like protein 1 concentrations in the cerebrospinal fluid of patients with mild cognitive impairment as a dynamic biomarker of Alzheimer's disease. *J. Alzheimers Dis.* **43**, 1031-1037 (2015).

43. Tarawneh, R. *et al.* Cerebrospinal Fluid Markers of Neurodegeneration and Rates of Brain Atrophy in Early Alzheimer Disease. *JAMA Neurol.* **72**, 656-665 (2015).

44. Babic, L. M., Borovecki, F., Dejanovic, N., Hof, P. R., Simic, G. Predictive Value of Cerebrospinal Fluid Visinin-Like Protein-1 Levels for Alzheimer's Disease Early Detection and Differential Diagnosis in Patients with Mild Cognitive Impairment. *J. Alzheimers Dis.* **50**, 765–778 (2016).

45. Kester, M. I. *et al.* Cerebrospinal fluid VILIP-1 and YKL-40, candidate biomarkers to diagnose, predict and monitor Alzheimer's disease in a memory clinic cohort. *Alzheimers Res. Ther.* **7**, 59 (2015).

46. Jack, C. R., Jr. *et al.* NIA-AA Research Framework: Toward a biological definition of Alzheimer's disease. *Alzheimers Dement.* **14**, 535-562 (2018).

47. Simonsen, A. H. *et al.* Novel panel of cerebrospinal fluid biomarkers for the prediction of progression to Alzheimer dementia in patients with mild cognitive impairment. *Arch. Neurol.* **64**, 366-370 (2007).

48. Janelidze, S. *et al.* CSF biomarkers of neuroinflammation and cerebrovascular dysfunction in early Alzheimer disease. *Neurology.* **91**, e867-e877 (2018).

49. Nordengen, K. *et al.* Glial activation and inflammation along the Alzheimer's disease continuum. *J. Neuroinflammation.* **16**, 46 (2019).

50. Westin, K. *et al.* CCL2 is associated with a faster rate of cognitive decline during early stages of Alzheimer's disease. *PLoS One.* **7**, e30525 (2012).

51. Suárez-Calvet, M. *et al.* CSF progranulin increases in the course of Alzheimer's disease and is associated with sTREM2, neurodegeneration and cognitive decline. *EMBO Mol. Med.* **10** (2018).

52. Comi, C. *et al.* Osteopontin is increased in the cerebrospinal fluid of patients with Alzheimer's disease and its levels correlate with cognitive decline. *J. Alzheimers Dis.* **19**, 1143-1148 (2010).

53. Heywood, W. E. *et al.* Identification of novel CSF biomarkers for neurodegeneration and their validation by a high-throughput multiplexed targeted proteomic assay. *Mol. Neurodegener.* **10**, 64 (2015).

54. Sun, Y. *et al.* Elevated osteopontin levels in mild cognitive impairment and Alzheimer's disease. *Mediators Inflamm.* **2013**, 615745 (2013).

55. Paterson, R. W. *et al.* A targeted proteomic multiplex CSF assay identifies increased malate dehydrogenase and other neurodegenerative biomarkers in individuals with Alzheimer's disease pathology. *Transl. Psychiatry.* **6**, e952 (2016).

56. Shi, M. *et al.* Cerebrospinal fluid peptides as potential Parkinson disease biomarkers: a staged pipeline for discovery and validation. *Mol. Cell Proteomics.* **14**, 544-555 (2015).

57. Bornsen, L., Khademi, M., Olsson, T., Sorensen, P. S. & Sellebjerg, F. Osteopontin concentrations are increased in cerebrospinal fluid during attacks of multiple sclerosis. *Mult Scler.* **17**, 32-42 (2011).

58. Heslegrave, A. *et al.* Increased cerebrospinal fluid soluble TREM2 concentration in Alzheimer's disease. *Mol. Neurodegener.* **11**, 3 (2016).

59. Piccio, L. *et al.* Cerebrospinal fluid soluble TREM2 is higher in Alzheimer disease and associated with mutation status. *Acta Neuropathol.* **131**, 925-933 (2016).

60. Brosseron, F. *et al.* Characterization and clinical use of inflammatory cerebrospinal fluid protein markers in Alzheimer's disease. *Alzheimers Res. Ther.* **10**, 25 (2018).

61. Heywood, W. E. *et al.* CSF pro-orexin and amyloid-beta38 expression in Alzheimer's disease and frontotemporal dementia. *Neurobiol. Aging.* **72**, 171-176 (2018).

62. Suárez-Calvet, M. *et al.* Early increase of CSF sTREM2 in Alzheimer's disease is associated with tau related-neurodegeneration but not with amyloid-beta pathology. *Mol. Neurodegener.* **14**, 1 (2019).

63. Henjum, K. *et al.* Cerebrospinal fluid soluble TREM2 in aging and Alzheimer's disease. *Alzheimers Res. Ther.* **8**, 17 (2016).

64. Gispert, J. D. *et al.* The APOE epsilon4 genotype modulates CSF YKL-40 levels and their structural brain correlates in the continuum of Alzheimer's disease but not those of sTREM2. *Alzheimers Dement (Amst).* **6**, 50-59 (2017).

65. Suárez-Calvet, M. *et al.* sTREM2 cerebrospinal fluid levels are a potential biomarker for microglia activity in early-stage Alzheimer's disease and associate with neuronal injury markers. *EMBO Mol. Med.* **8**, 466-476 (2016).

66. Suárez-Calvet, M. *et al.* Early changes in CSF sTREM2 in dominantly inherited Alzheimer's disease occur after amyloid deposition and neuronal injury. *Sci. Transl. Med.* **8**, 369ra178 (2016).

67. Rauchmann, B. S., Schneider-Axmann, T., Alexopoulos, P. & Perneczky, R. CSF soluble TREM2 as a measure of immune response along the Alzheimer's disease continuum. *Neurobiol. Aging.* **74**, 182-190 (2019).

68. Ohara, T. *et al.* Serum Soluble Triggering Receptor Expressed on Myeloid Cells 2 as a Biomarker for Incident Dementia: The Hisayama Study. *Ann. Neurol.* **85**, 47-58 (2019).

69. Craig-Schapiro, R. *et al.* YKL-40: a novel prognostic fluid biomarker for preclinical Alzheimer's disease. *Biol. Psychiatry.* **68**, 903-912 (2010).

70. Mattsson, N. *et al.* Cerebrospinal fluid microglial markers in Alzheimer's disease: elevated chitotriosidase activity but lack of diagnostic utility. *Neuromolecular Med.* **13**, 151-159 (2011).

71. Wildsmith, K. R. *et al.* Identification of longitudinally dynamic biomarkers in Alzheimer's disease cerebrospinal fluid by targeted proteomics. *Mol. Neurodegener.* **9**, 22 (2014).

72. Alcolea, D. *et al.* Amyloid precursor protein metabolism and inflammation markers in preclinical Alzheimer disease. *Neurology.* **85**, 626-633 (2015).

73. Alcolea, D. *et al.* Elevated YKL-40 and low sAPPbeta:YKL-40 ratio in antemortem cerebrospinal fluid of patients with pathologically confirmed FTLD. *J. Neurol. Neurosurg. Psychiatry.* **90**, 180-186 (2019).

74. Wennstrom, M. *et al.* The Inflammatory Marker YKL-40 Is Elevated in Cerebrospinal Fluid from Patients with Alzheimer's but Not Parkinson's Disease or Dementia with Lewy Bodies. *PLoS One.* **10**, e0135458 (2015).

75. Gispert, J. D. *et al.* CSF YKL-40 and pTau181 are related to different cerebral morphometric patterns in early AD. *Neurobiol. Aging.* **38**, 47-55 (2016).

76. Choi, J., Lee, H. W. & Suk, K. Plasma level of chitinase 3-like 1 protein increases in patients with early Alzheimer's disease. *J. Neurol.* **258**, 2181-2185 (2011).

77. Villar-Pique, A. *et al.* Plasma YKL-40 in the spectrum of neurodegenerative dementia. *J. Neuroinflammation.* **16**, 145 (2019).

78. Toledo, J. B. *et al.* CSF Apo-E levels associate with cognitive decline and MRI changes. *Acta Neuropathol.* **127**, 621-632 (2014).

79. Johansson, P. *et al.* Reduced Cerebrospinal Fluid Concentration of Apolipoprotein A-I in Patients with Alzheimer's Disease. *J. Alzheimers Dis.* **59**, 1017-1026 (2017).

80. Desikan, R. S. *et al.* Heart fatty acid binding protein and Abeta-associated Alzheimer's neurodegeneration. *Mol. Neurodegener.* **8**, 39 (2013).

81. Guo, L. H., Alexopoulos, P. & Perneczky, R. Heart-type fatty acid binding protein and vascular endothelial growth factor: cerebrospinal fluid biomarker candidates for Alzheimer's disease. *Eur. Arch. Psychiatry Clin. Neurosci.* **263**, 553-560 (2013).

82. Olsson, B. *et al.* Cerebrospinal fluid levels of heart fatty acid binding protein are elevated prodromally in Alzheimer's disease and vascular dementia. *J. Alzheimers Dis.* **34**, 673-679 (2013).

83. Leung, Y. Y. *et al.* Identifying amyloid pathology-related cerebrospinal fluid biomarkers for Alzheimer's disease in a multicohort study. *Alzheimers Dement (Amst).* **1**, 339-348 (2015).

84. Chiasserini, D. *et al.* Differential role of CSF fatty acid binding protein 3, alpha-synuclein, and Alzheimer's disease core biomarkers in Lewy body disorders and Alzheimer's dementia. *Alzheimers Res. Ther.* **9**, 52 (2017).

85. Deming, Y. *et al.* A potential endophenotype for Alzheimer's disease: cerebrospinal fluid clusterin. *Neurobiol. Aging.* **37**, 208.e201-208.e209 (2016).

86. Hye, A. *et al.* Plasma proteins predict conversion to dementia from prodromal disease. *Alzheimers Dement.* **10**, 799-807 (2014).

87. Schmidt, F. M. *et al.* Cerebrospinal fluid melanin-concentrating hormone (MCH) and hypocretin-1 (HCRT-1, orexin-A) in Alzheimer's disease. *PLoS One.* **8**, e63136 (2013).

88. Gabelle, A. *et al.* Cerebrospinal fluid levels of orexin-A and histamine, and sleep profile within the Alzheimer process. *Neurobiol. Aging.* **53**, 59-66 (2017).

89. Osorio, R. S. *et al.* Orexin-A is Associated with Increases in Cerebrospinal Fluid Phosphorylated-Tau in Cognitively Normal Elderly Subjects. *Sleep.* **39**, 1253-1260 (2016).

90. Schultz, K. *et al.* Transthyretin as a potential CSF biomarker for Alzheimer's disease and dementia with Lewy bodies: effects of treatment with cholinesterase inhibitors. *Eur. J. Neurol.* **17**, 456-460 (2010).

91. Cordero-Llana, O. *et al.* Clusterin secreted by astrocytes enhances neuronal differentiation from human neural precursor cells. *Cell Death Differ.* **18**, 907-913 (2011).

92. Velayudhan, L. *et al.* Plasma transthyretin as a candidate marker for Alzheimer's disease. *J. Alzheimers Dis.* **28**, 369–375 (2012).
